# Remodeling of the Intra‐Conduit Inflammatory Microenvironment to Improve Peripheral Nerve Regeneration with a Neuromechanical Matching Protein‐Based Conduit

**DOI:** 10.1002/advs.202302988

**Published:** 2024-03-02

**Authors:** Jia‐Yi Wang, Ya Yuan, Shu‐Yan Zhang, Shun‐Yi Lu, Guan‐Jie Han, Meng‐Xuan Bian, Lei huang, De‐Hua Meng, Di‐Han Su, Lan Xiao, Yin Xiao, Jian Zhang, Ning‐Ji Gong, Li‐Bo Jiang

**Affiliations:** ^1^ Department of Orthopaedic Surgery Zhongshan Hospital Fudan University Shanghai 200032 China; ^2^ Department of Rehabilitation Zhongshan Hospital Fudan University Shanghai 200032 China; ^3^ The Key Laboratory for Ultrafine Materials of Ministry of Education, Engineering Research Centre for Biomedical Materials of Ministry of Education Frontiers Science Center for Materiobiology and Dynamic Chemistry School of Materials Science and Engineering East China University of Science and Technology Shanghai 200237 China; ^4^ School of Mechanical Medical and Process Engineering Centre for Biomedical Technologies Queensland University of Technology Brisbane 4059 Australia; ^5^ Australia‐China Centre for Tissue Engineering and Regenerative Medicine Queensland University of Technology Brisbane 4059 Australia; ^6^ School of Medicine and Dentistry & Menzies Health Institute Queensland Griffith University Gold Coast 4222 Australia; ^7^ Department of Emergency Department of Orthopedics The Second Hospital Cheeloo College of Medicine Shandong University Jinan Shandong 250033 China

**Keywords:** electrical stimulation, intra‐conduit inflammatory microenvironment, neuromechanical matching, peripheral nerve regeneration, silk fibroin

## Abstract

Peripheral nerve injury (PNI) remains a challenging area in regenerative medicine. Nerve guide conduit (NGC) transplantation is a common treatment for PNI, but the prognosis of NGC treatment is unsatisfactory due to 1) neuromechanical unmatching and 2) the intra‐conduit inflammatory microenvironment (IME) resulting from Schwann cell pyroptosis and inflammatory‐polarized macrophages. A neuromechanically matched NGC composed of regenerated silk fibroin (RSF) loaded with poly(3,4‐ethylenedioxythiophene): poly(styrene sulfonate) (P:P) and dimethyl fumarate (DMF) are designed, which exhibits a matched elastic modulus (25.1 ± 3.5 MPa) for the peripheral nerve and the highest 80% elongation at break, better than most protein‐based conduits. Moreover, the NGC can gradually regulate the intra‐conduit IME by releasing DMF and monitoring sciatic nerve movements via piezoresistive sensing. The combination of NGC and electrical stimulation modulates the IME to support PNI regeneration by synergistically inhibiting Schwann cell pyroptosis and reducing inflammatory factor release, shifting macrophage polarization from the inflammatory M1 phenotype to the tissue regenerative M2 phenotype and resulting in functional recovery of neurons. In a rat sciatic nerve crush model, NGC promoted remyelination and functional and structural regeneration. Generally, the DMF/RSF/P:P conduit provides a new potential therapeutic approach to promote nerve repair in future clinical treatments.

## Graphical abstract

In this study, a novel mechanical matching DMF/RSF/P:P conduit based on RSF and bioencapsulated PEDOT:PSS is designed and developed, which can inhibit Schwann cell pyroptosis and induce macrophage polarization toward the M2 subtype to improve peripheral nerve regeneration. The current study shows a promising strategy to promote peripheral nerve regeneration by remodeling the intraconduit IME at the nerve injury site.

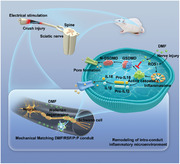

## 1 Introduction

Peripheral nerve injury (PNI) is a common clinical condition that can result in severe disability and a significant burden on patients and society.^[^ ^1^ ^]^ The main causes of the poor prognosis of PNI treatment are interference from surrounding muscle tissue and inflammation.^[^ ^2^ ^]^ The main strategy for PNI treatment via nerve guide conduits (NGCs) is to provide ideal mechanical support for nerve regeneration and prevent peripheral tissues from interfering with nerve repair. Although NGCs can block peripheral inflammatory cell invasion, the inflammatory microenvironment (IME) in NGCs can inhibit the functional recovery of Schwann cells and neurons.

The IME comprises inflammatory cells, proinflammatory enzymes, and inflammatory mediators.^[^ ^3^ ^]^ At nerve injury sites, the main inflammatory cells are proinflammatory macrophages and dysfunctional Schwann cells. During nerve injury, Schwann cells undergo pyroptosis and release several inflammatory factors, such as interleukin (IL)−18 and IL‐1β.^[^ ^4^ ^]^ Moreover, M1 macrophages secrete tumor necrosis factor α, which ultimately inhibits PNI regeneration directly. Studies have shown that the micropatterning^[^ ^5^ ^]^ and topology^[^ ^6^ ^]^ of NGCs can modulate macrophage polarization, but NGCs cannot inhibit Schwann cell pyroptosis or reduce the release of inflammatory factors in conduits. Dimethyl fumarate (DMF; Tecfidera) is a chemical approved by the US Food and Drug Administration for treating multiple sclerosis.^[^ ^7^ ^]^ Previous studies have shown that DMF can promote the polarization of macrophages to the M2 subtype and inhibit pyroptosis by targeting Gasdermin D (GSDMD).^[^ ^8^ ^]^ Therefore, the local application of the DMF slow‐release system could be an effective complement to NGCs for regulating the IME.

Many natural or artificial materials have been used to synthesize NGCs.^[^ ^9^ ^]^ However, the treatment efficacy of silk fibroin‐based NGCs is unsatisfactory due to their unmatched mechanical properties.^[^ ^10^ ^]^ Mechanically matched NGCs are an essential component of tissue engineering and tissue regeneration.^[^ ^11^ ^]^ The elastic modulus and elongation at the break of the human sciatic nerve are approximately 6.71 ± 0.93 MPa and 30.28% ± 3.19%, respectively.^[^ ^12^ ^]^ Some NGCs with several hundred MPa or GPa elastic moduli are stiffer than nerves,^[^ ^13^ ^]^ which might cause nerve entrapment and even hinder nerve regeneration.^[^ ^14^ ^]^ Moreover, some NGCs with very low elastic moduli cannot support PNRs and may collapse after transplantation, resulting in nerve compression by the surrounding muscles and impeding nerve regeneration.^[^ ^15^ ^]^ In addition, the low elongation at break might cause NGCs to break and fall off during nerve movement, leading to difficulty in meeting the needs of long‐term application.^[^ ^16^ ^]^ Recently, regenerated silk fibroin (RSF)–based materials have been widely used in regenerative medicine fields due to their remarkable biocompatibility,^[^ ^17^ ^]^ such as cartilage,^[^ ^18^ ^]^ nerve, and cornea repair.^[^ ^19^ ^]^ Currently, silk fibroin (SF)–based conduits have been manufactured for nerve repair, and NGCs for peripheral nerve regeneration (PNR) should have a neutral modulus of elasticity and high elongation at break. A high or low elastic modulus is detrimental to PNR.^[^ ^20^ ^]^ Furthermore, electrical stimulation (ES) can treat PNI by enhancing the extension of nerve axons, promoting neurotrophic factor synthesis, and regulating macrophage polarization.^[^ ^21^ ^]^ Currently, the effect of electrical conduction is enhanced by the addition of conductive substances, such as polypyrrole, polyaniline, and polythiophene, to NGCs.^[^ ^22^ ^]^ However, the biocompatibility of these materials is unsatisfactory.^[^ ^23^ ^]^ Poly(3,4‐ethylenedioxythiophene):poly(styrene sulfonate) (PEDOT:PSS) is an excellent metal and inorganic semiconductor. It possesses excellent conductivity and biocompatibility and shows good stability in various environments.^[^ ^24^ ^]^

**Scheme 1:**
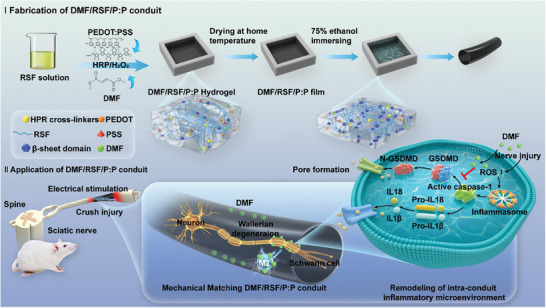
Scheme showing the fabrication and application of multifunctional conduits for peripheral nerve regeneration. I) Fabrication process of DMF/RSF/P:P conduits. II) Concept and mechanisms of DMF/RSF/P:P conduits combined with electrical stimulation (ES) to alleviate the inflammatory response in the microenvironment at nerve injury sites by inducing macrophage polarization toward the M2 subtype and inhibiting Schwann cell pyroptosis.

To promote PNR, we designed and manufactured an easy‐to‐make DMF/RSF/P:P composite NGC with high flexibility, piezoresistive properties, slow‐release properties, and neuromechanical matching (**Scheme** **1**). We evaluated the cytocompatibility, optimal PEDOT:PSS, and DMF concentration in vitro. In addition, the DMF/RSF/P:P composite combined with ES inhibited Schwann cell pyroptosis and induced macrophage polarization toward the M2 subtype both in vitro and in vivo, thereby remodeling the IME inside the conduits at the injury sites and promoting the recovery of injured neurons. Notably, DMF/RSF/P:P NGCs with ES were applied to a Sprague–Dawley (SD) rat sciatic nerve crush model to evaluate the morphology and sensory and motor functions of sciatic nerves, and electrophysiology and immunohistochemical analyses were performed to assess therapeutic efficacy.

## 2 Results and Discussion

### 2.1 Morphology and Structure of DMF/RSF/P:P Conduits

**Figure 1:**
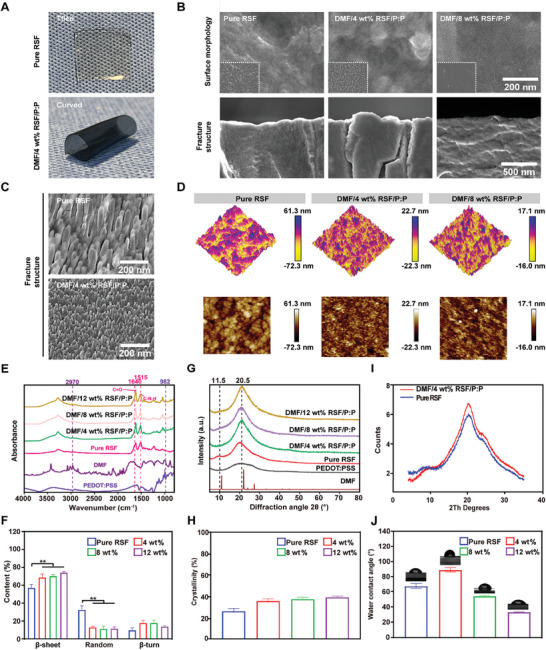
Morphology and structure of DMF/RSF/P:P conduits. A) Macrograph of a pure RSF in the tiled state and a DMF/4 wt% RSF/P:P conduit in the curved state. B) Scanning electron microscopy images of DMF/RSF/P:P conduits with different RSF concentrations. C) Cryo‐SEM images of pure RSF and DMF/RSF/P:P conduits (4 wt.% RSF). D) AFM image of DMF/RSF/P:P conduits with different RSF concentrations. E) FT‐IR spectra of DMF/RSF/P:P conduits with different RSF concentrations. F) Fitting analysis of different conduits based on the amide I band in the FT‐IR spectra. G) XRD patterns of the different conduit samples. H) Deconvolution analysis of the XRD curves. I) WAXS results of Pure RSF and DMF/RSF/P:P conduits. J) Contact angles of DMF/RSF/P:P conduits. Figure 1F was analyzed using one‐way ANOVA followed by Tukey's post hoc test and are presented as the means ± SDs. ^**^ *p* < 0.01 compared to the Pure RSF group. ^*^ *p* < 0.05 compared to the Pure RSF group. *n* = 6. P:P, PEDOT:PSS.

Transparent Pure RSF (4 wt.%) conduits have poor mechanical properties and cannot be rolled or sutured (**Figure** **1**A). However, DMF/4 wt.% RSF/P:P conduits with a blue‐black color from the conductive component PEDOT:PSS can be easily formed into a tube for nerve wrapping. Figure 1B shows the scanning electron microscopy (SEM) images of DMF/RSF/P:P conduits with different RSF concentrations (4 and 8 wt.%), where the surface roughness of the conduit materials decreases with increasing RSF concentration. A very dense surface structure and grainy surface roughness were observed for the 8 and 4 wt.% RSFs, respectively, which were conducive to cell adhesion. After breaking using liquid nitrogen, more bulges and rough structures can be observed at the conduit cross‐section in the Pure RSF and DMF/4 wt.% RSF/P:P groups than in the DMF/8 wt.% RSF/P:P group (Figure 1B), indicating that the conduit materials in the DMF/8 wt.% RSF/P:P group were more compact, which was attributed to the increased RSF concentration. Moreover, Figure 1C shows the fracture structure cryo‐SEM images of the Pure RSF and DMF/4 wt.% RSF/P:P films, wherein a dense columnar structure can be observed in both the Pure RSF and DMF/4 wt.% RSF/P:P. Moreover, the granular objects on the surface differed in the latter columnar structures, which may be due to the addition of PEDOT:PSS or DMF. Figure 1D shows atomic force microscopy (AFM) images of DMF/RSF/P:P conduits with different RSF concentrations. The surface roughness (Ra) of the Pure RSF group was significantly greater than that of the other DMF/RSF/P:P groups, and the roughness parameter Ra of the conduits decreased with increasing RSF concentration, which was consistent with the data obtained from the surface roughness tester (Figure S1, Supporting Information).

Fourier transform infrared spectroscopy (FT‐IR) and X‐ray diffraction (XRD) were performed to characterize the conduit structure. Figure 1E shows the presence of β‐sheet domains in silk fibroin in the FT‐IR spectra at 1265, 1515, and 1640 cm^−1^.^[^ ^25^ ^]^ The strong bands at 1515 and 1640 cm^−1^ were attributed to C‐N‐H bending and C═O stretching vibrations, respectively.^[^ ^10^ ^]^ The band at 982 cm^−1^ was attributed to the C‐O‐C symmetric stretching vibration in PEDOT:PSS,^[^ ^26^ ^]^ and the band at 2970 cm^−1^ was ascribed to the CH_3_ asymmetric stretching vibration corresponding to DMF.^[^ ^27^ ^]^ Most importantly, the intensity of the β‐sheet peak (1265, 1515, and 1640 cm^−1^) increased in the DMF/RSF/P:P samples compared with that in the Pure RSF group, which could be due to the random coil structure in the DMF/RSF/P:P conduits being transformed into β‐sheet domains after treatment with horseradish peroxidase (HRP)/H_2_O_2_ and ethanol immersion. Furthermore, we performed a deconvolution analysis of the amide I band in the FT‐IR spectra following previous methods in the literature (Figure S2, Supporting Information).^[^ ^28^ ^]^ The β‐sheet domain content in the DMF/RSF/P:P conduits with HRP/H_2_O_2_ enzymatic cross‐linking and ethanol induction was greater (≈70%) than that in the Pure RSF group (57.32% ± 3.7%) (Figure 1F). Figure 1G shows the XRD pattern results. Compared with that of the Pure RSF group, the diffraction peak of the DMF/RSF/P:P conduit was sharper (20.5°) due to the increase in β‐sheet crystallites in silk fibroin. Moreover, the diffraction peak (11.5°) in the Pure RSF conduit is more obvious than that in the DMF/RSF/P:P conduit. Moreover, the deconvolution analysis of the XRD curves for each group (Figure S3, Supporting Information) showed that the crystallinity of the RSF conduits in the DMF/RSF/P:P groups (≈40%) was greater than that in the Pure RSF group (25.87% ± 3.6%) (Figure 1H), which was consistent with the FT‐IR results. Notably, the crystallinity of the RSF conduits increased with increasing RSF concentration. Furthermore, wide‐angle X‐ray scattering (WAXS) was used to further analyze the β‐sheet domains (Figure 1I). The Debye–Scherrer equation D = Kλ/(βcosθ) showed that the β‐sheet structural domains in DMF/RSF/P:P conduits were smaller (1.9 nm) than those in Pure RSF conduits (2.0 nm),^[^ ^29^ ^]^ which was mainly because the HRP pre‐crosslinking network greatly restricts RSF chain movement and organization during β‐sheet structure formation; thus, the crystal size of the β‐sheet domains was limited. Previous studies have shown that the size of β‐sheet structural domains could significantly affect the mechanical properties of materials.^[^ ^30^ ^]^

In addition, we also examined the contact angles of the DMF/RSF/P:P conduits with different RSF concentrations (Figure 1J). Moreover, the surface contact angle and material hydrophilicity increased with decreasing RSF concentration. The contact angle was 89.2° ± 2.9° in the DMF/4 wt% RSF/P:P conduit compared with that in the DMF/8 wt% RSF/P:P (54.6° ± 0.8°) and DMF/12 wt.% RSF/P:P conduits (33.7° ± 0.5°).

### 2.2 Multifunctional Properties of DMF/RSF/P:P Conduits

**Figure 2:**
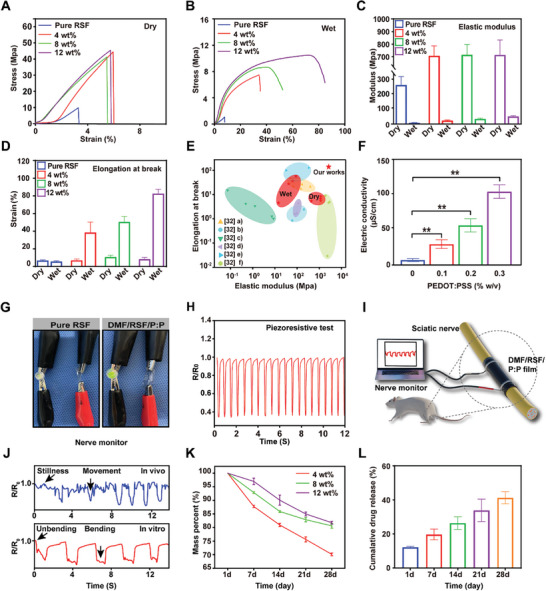
Multifunctional properties of DMF/RSF/P:P conduits. Stress‐strain curves of pure RSF conduits and DMF/RSF/P:P composites in the dry A) and wet B) states. C) The elastic modulus of Pure RSF conduits and DMF/RSF/P:P composites with different RSF concentrations in the dry and wet states. D) Elongation at break of DMF/RSF/P:P composites with different RSF concentrations in the dry and wet states. E) Ashby plot with elongation at break versus elastic modulus, where the red area represents the DMF/RSF/P:P conduit. F) Electrical conductivity of conduits with different PEDOT:PSS concentrations. G) Electrically conductive paths made of pure RSF conduits and DMF/RSF/P:P composites for LED illumination. H) Piezoresistive test of DMF/RSF/P:P composites. I) Schematic diagram of DMF/RSF/P:P conduits monitoring sciatic nerve movements. J) Results of DMF/RSF/P:P conduits monitoring sciatic nerve movements in vivo and in vitro. K) Degradation behavior of DMF/RSF/P:P composites in vitro. L) Cumulative DMF release results for DMF/4 wt.% RSF/P:P conduits after 28 days were determined by using HPLC in vitro. Figure 2F was analyzed using one‐way ANOVA followed by Tukey's post hoc test and are presented as the means ± SDs. ^**^ *p* < 0.01, ^*^ *p* < 0.05, and ^**^ indicate statistical significance between the indicated groups; *n* = 6. HPLC, high‐performance liquid chromatography.

**Figure** **2**A–E shows the mechanical properties of the DMF/RSF/P:P conduits. Figure S4 (Supporting Information) shows the stretching process of the DMF/RSF/P:P conduits in a wet state. Figure 2A–B shows the stress‐strain curves of Pure RSF and DMF/RSF/P:P conduits with different RSF concentrations in dry and wet states, respectively. The curve of the DMF/RSF/P:P conduit in the dry state was a straight line, showing the typical characteristics of brittle fracture. In contrast, the yield point and yield plateau appeared during the stretching process of the DMF/RSF/P:P conduit in the wet state and showed typical characteristics of ductile fracture. Moreover, this phenomenon indicates that water plays a role in the plasticization of the silk conduit. Moreover, pure RSF conduits in the wet state exhibited low elongation at break (5.8% ± 0.5%) and elastic modulus (8.5 ± 1.3 MPa), and HRP cross‐linking greatly improved the mechanical properties of the conduit materials. The elastic modulus of the DMF/RSF/P:P conduits in the dry state was greater than that in the wet state (Figure 2C), indicating that the DMF/RSF/P:P conduits in the dry state were rigid and brittle, whereas the DMF/RSF/P:P conduits in the wet state were flexible and soft, which was consistent with the findings of previous studies.^[^ ^31^ ^]^ The elastic modulus in the wet state increased with increasing RSF concentration, which could be due to the increase in the amount of RSF inter cross‐linkers with enzymatic cross‐linking and beta‐sheet cross‐linking. The elastic modulus of optimal NGCs for peripheral nerve regeneration should be slightly greater than that of the nerve to provide support and prevent the surrounding muscles from compressing the sciatic nerve. Subsequently, the elastic modulus of the conduit material gradually decreases with degradation in the later stage of nerve repair, which is more conducive to the free movement of the nerve. The elastic modulus of the DMF/4 wt.% RSF/P:P conduit (25.1 ±3.5 MPa) was the most similar to that of the sciatic nerve. Thus, 4 wt.% RSF was selected for use in animal models. The elongation at break in DMF/RSF/P:P conduits in the dry state was lower than that in the wet state and increased with increasing RSF concentration in the wet state (Figure 2D). The elongation at break of 4 wt.% RSF in the wet state reached 38%, which was sufficient to meet the soft and elastic requirements for wrapping the sciatic nerve. Moreover, the elongation at break of DMF/12 wt.% RSF/P:P conduits can reach approximately 80%, eight times that of Pure RSF conduits, which may be due to the crucial role played by the size and distribution of β‐sheet structural domains in DMF/RSF/P:P conduits in mechanical improvement. Figure 2E shows the Ashby plot with elongation at break versus elastic modulus, and the red area represents the DMF/RSF/P:P conduit. Compared with other silk fibroins as main component‐based materials,^[^ ^32^ ^]^ the DMF/RSF/P:P conduits showed a matching elastic modulus and greater elongation at break, which can be better matched to nerves.

To the best of our knowledge, the positive role of ES in PNI regeneration is clear.^[^ ^33^ ^]^ Figure S5A shows digital photographs of pre‐conduit solutions containing different concentrations of the conductive component PEDOT:PSS. The electrical conductivity of the DMF/RSF/P:P conduits increased to approximately 130 µS cm^−1^ when the PEDOT:PSS content reached 0.3% w v^−1^ (Figure 2F), indicating the favorable conductivity of the DMF/RSF/P:P conduits, which was consistent with the results of previous studies.^[^ ^34^ ^]^ An illuminated light‐emitting diode (LED) was observed, particularly in a circuit connected to conduits with high PEDOT:PSS concentrations (0.3% w v^−1^) (Figure 2G). Moreover, we tested the potential of DMF/RSF/P:P conduits as flexible and stretchable sensors. The sensor response was evaluated using R/R_0_ × 100%, where R/R_0_ is the ratio of the instantaneous resistance at a given strain to the initial resistance at zero strain, R_0_ is the initial resistance, and R is the real‐time resistance.^[^ ^35^ ^]^ Figure 2H shows the regular and repeatable electrical signals after stress deformation of the conduits, including the piezoresistive test, indicating that the conduits are sensitive to changes in stress deformation. Therefore, we verified that the conduits could detect pressure changes caused by tiny movements, such as sciatic nerve movements (Figure 2I,J), fist‐making (Figure S5B, Supporting Information), and pressing (Figure S5C, Supporting Information). Figure 2I shows that DMF/RSF/P:P is used to monitor pressure changes due to muscle contraction in vivo in the rat sciatic nerve. The results (Figure 2J) showed that the electrical signals of the rats were significantly different during motion or at rest; therefore, the electrical signals served as a monitoring function for the rat sciatic nerve. In addition, DMF/RSF/P:P was used to monitor pressure due to changes in bending and unbending in vitro in the rat sciatic nerve, and the results showed that the electrical signals in the two states were different. Therefore, DMF/RSF/P:P conduits can be applied as ideal sensors for monitoring shape changes. Notably, we also verified the conduit stability, a crucial indicator for evaluating the sensor (Figure S5D, Supporting Information).^[^ ^36^ ^]^

Degradability is an essential property affecting the application of electroactive biomaterials in vivo.^[^ ^37^ ^]^ Proteinase XIV is a non‐mammalian enzyme commonly used to study the degradation of silk materials in vitro.^[^ ^38^ ^]^ To examine degradation, DMF/RSF/P:P conduits containing different RSF concentrations were soaked in 0.1 U mL^−1^ protease XIV solution. The mass loss of the conduits was quantified, and the conduits containing 4 wt% RSF had greater weight loss than those containing other RSF concentrations with increasing treatment time in protease XIV (Figure 2K). A higher β‐sheet domain content and crystallite size decrease the degradation rate of silk protein materials, which may be due to the difficulty in penetrating the conduit with protease XIV solution, which has a higher crystallite ratio and smaller contact area; these findings were reported previously^[^ ^22^, ^39^ ^]^ and are consistent with previous FT‐IR and XRD results. After 28 days of exposure to protease XIV solution, the weight loss was >30%, indicating good biodegradability of the DMF/4 wt% RSF/P:P conduits. Furthermore, Figure S6A shows the specific elastic modulus of the DMF/4 wt% RSF/P:P conduits at the corresponding time points (7, 14, 21, and 28 days). The results indicate that as degradation progresses, the elastic modulus of the conduits decreases. On day 28, the elastic modulus of the conduits reaches approximately half of the original elastic modulus. In addition, the in vivo degradation characteristics of DMF/4 wt.% RSF/P:P conduits and their mechanical properties at the corresponding time points were also tested (Figure S6B,C, Supporting Information). The results showed that the rate of in vivo degradation of DMF/4 wt.% RSF/P:P conduits was slower than that in vitro, possibly due to the lower concentration of degrading enzymes in vivo. The results indicate that the elastic modulus of DMF/4 wt.% RSF/P:P conduits after 90 days (7.82 ± 3.97 MPa) of in vivo degradation is sufficient to support the requirements of nerve regeneration (6.71 ± 0.93 MPa).^[^ ^12^ ^]^

To date, many excellent biomaterial systems, such as hydrogel systems, that can effectively deliver cells or therapeutic agents have been used to promote nerve regeneration.^[^ ^40^ ^]^ However, compared to current biomaterial systems, most hydrogel materials degrade rapidly and degrade completely within 2 weeks or even less, making it difficult to provide sufficient mechanical support for peripheral nerve regeneration.^[^ ^41^ ^]^ Moreover, some hydrogel systems have poor mechanical properties, making it difficult to provide effective mechanical support for nerve regeneration. For example, the elastic modulus of some hydrogels is in the KPa range, which is significantly lower than the elastic modulus of nerves.^[^ ^40^ ^]^ Several 3D‐printed biodelivery systems that can effectively deliver cells are also widely used to promote peripheral nerve regeneration, but their complex preparation methods limit their widespread application.^[^ ^42^ ^]^

High‐performance liquid chromatography (HPLC) was used to quantify the cumulative release of DMF,^[^ ^43^ ^]^ which was accompanied by the degradation of DMF/RSF/P:P conduits treated with protease XIV solution in vitro (Figure 2L). Moreover, >30% DMF was released after 28 days of exposure to the protease XIV solution, suggesting favorable drug release properties that can ensure a sufficient amount of the released drug for early‐stage nerve repair.

### 2.3 Biological Assessment in Vitro

#### 2.3.1 Cell Viability and Cell Adhesion

Generally, increased PEDOT:PSS concentrations in DMF/RSF/P:P conduits increase conduit conductivity, but high PEDOT:PSS concentrations may affect cell viability. Cell viability was assessed using the Cell Counting Kit‐8 (CCK‐8) assay. The viability of Schwann cells in DMF/RSF/P:P conduits decreased from days 1 to 3 when the PEDOT:PSS concentration reached 0.3% w v^−1^ on days 2 and 3, indicating that high concentrations of PEDOT:PSS may be toxic to Schwann cells (Figure S7A, Supporting Information). In addition, live–dead staining results indicated that the number of dead Schwann cells significantly increased at high PEDOT:PSS concentrations on days 1 (Figure S7B, Supporting Information), 3 (Figure S7C, Supporting Information), and 7 (Figure S7D, Supporting Information). The cell adhesion properties of the DMF/RSF/P:P conduits were unsatisfactory (Figure S8A, Supporting Information), which was consistent with the findings of previous studies.^[^ ^23^ ^]^ Based on the previous contact angle results, the hydrophilicity of the DMF/4 wt.% RSF/P:P conduits generally negatively affects cell adhesion properties. In contrast, the rich PSS domain on the outer surface of the conduit, which has a negative charge, may also lead to difficulties in cell adhesion. To improve cell adhesion properties, surface modification with DMF/RSF/P:P conduits, such as the application of plasma, poly‐L‐lysine, or laminin, and an extracellular matrix could be effective.^[^ ^44^ ^]^ Our study showed that cell adhesion properties improved after the application of poly‐L‐lysine (Figure S8B, Supporting Information). Figure S9A (Supporting Information) shows the SEM images of the conduit in the presence of poly‐L‐lysine. Moreover, the FT‐IR results of the conduit in the presence of poly‐L‐lysine were not significantly different, indicating that the internal structure of the conduit was not affected after the addition of poly‐L‐lysine (Figure S9B, Supporting Information).

#### 2.3.2 DMF/RSF/P:P Conduits Combined with ES Attenuate the Inhibitory Effects of Schwann Cell Pyroptosis on PC12 Cell Function in Vitro

Certain DMF concentrations may have a protective effect on cells, but evidence shows that high DMF concentrations are toxic.^[^ ^45^ ^]^ The CCK‐8 assay was used to evaluate cell viability to optimize the concentration of DMF loaded with DMF/RSF/P:P conduits. From 0 to 48 h, the viability of Schwann cells in DMF/RSF/P:P conduits decreased when the DMF concentration reached 500 µm at 12, 24, and 48 h, indicating that DMF may be toxic to Schwann cells at high concentrations. Therefore, we used 400 µm DMF in subsequent experiments (Figure S10, Supporting Information).

**Figure 3:**
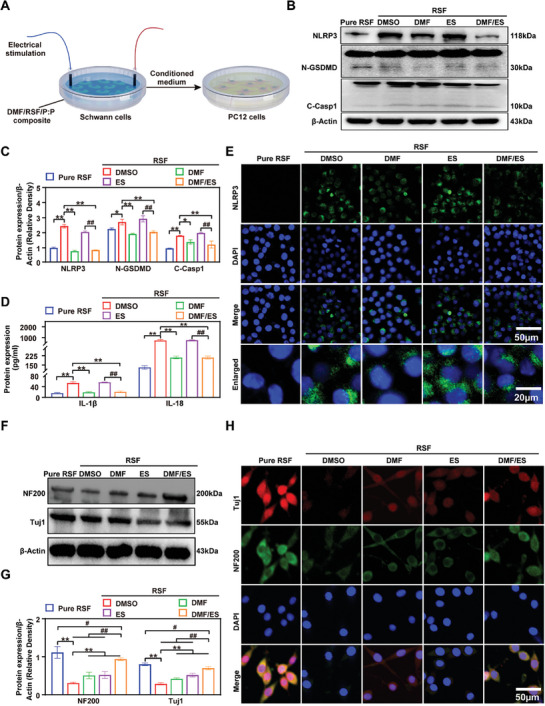
DMF/RSF/P:P conduits combined with ES attenuate the inhibitory effects of Schwann cell pyroptosis on PC12 cell function in vitro. A) Schematic diagram of DMF/RSF/P:P conduits combined with ES attenuating the inhibitory effects on PC12 cell function induced by Schwann cell pyroptosis. B,C) Protein levels and quantitative analysis of the pyroptosis‐related proteins NLRP3, N‐GSDMD, and C‐Casp1 in Schwann cells seeded in different conduit groups. β‐Actin was used as an internal control. D) IL‐18 and IL‐1β protein levels in culture supernatants of Schwann cells seeded in different conduit groups determined via ELISA. E) Immunofluorescence staining for NLRP3 (green) in Schwann cells seeded in different groups of membranes. (DAPI: blue). F,G) Protein levels and quantitative analysis of NF200 and Tuj1 in PC12 cells cultured in conditioned media obtained from Schwann cells seeded on different membranes. β‐Actin was used as an internal control. (H) Immunofluorescence staining for NF200 (green) and Tuj1 (red) in PC12 cells treated with Schwann cell‐conditioned media (DAPI: blue). Figure 3C,D,G were analyzed using one‐way ANOVA followed by Tukey's post hoc test and are presented as the means ± SDs. ^**^ *p* < 0.01 compared to the RSF/DMSO group. ^*^ < 0.05 compared to the RSF/DMSO group. ^##^ *p* < 0.01 compared to the RSF/DMF/ES group. ^#^ *p* < 0.05 compared to the RSF/DMF/ES group. *n* = 3.

Pyroptosis is a programmed cell death process characterized by continuous cell expansion until cell membrane rupture, resulting in the activation of a strong inflammatory response and the release of inflammatory factors, including IL‐18 and cytokines.^[^ ^46^ ^]^ Previous studies have reported that the release of IL‐18 and IL‐1β can impair neuronal function.^[^ ^47^ ^]^ Accordingly, SEM and transmission electron microscopy (TEM) were used to detect pyroptosis in Schwann cells after treatment with lipopolysaccharides (LPS)/adenosine triphosphate disodium (ATP) (Figure S11, Supporting Information). **Figure** **3**A shows a schematic diagram of DMF/RSF/P:P conduits combined with ES that attenuate the inhibitory effects on PC12 cell function induced by pyroptosis in Schwann cells. To explore the inhibitory effects of DMF/RSF/P:P conduits combined with ES on pyroptosis, Schwann cells were divided into five groups (the pure RSF, RSF/DMSO, RSF/DMF, RSF/ES, and RSF/DMF/ES groups). Except for those in the Pure RSF group, all the cells in all the groups were induced with LPS/ATP to establish a pyroptosis model. Compared with those in the RSF/DMSO group and RSF/ES group, the levels of the pyroptosis‐related proteins NOD‐like receptor family pyrin domain containing 3 (NLRP3), the N‐terminal fragment of GSDMD (N‐GSDMD), and cleaved cysteinyl aspartate‐specific protease‐1 (C‐Casp1) in the RSF/DMF and RSF/DMF/ES groups were significantly lower, indicating that the DMF/RSF/P:P conduits could effectively inhibit Schwann cell pyroptosis in vitro (Figure 3B,C). Furthermore, enzyme‐linked immunosorbent assay (ELISA) results showed that IL‐18 and IL‐1β release in the RSF/DMF and RSF/DMF/ES groups was significantly lower than that in the RSF/DMSO and RSF/ES groups (Figure 3D). Finally, immunofluorescence staining was used to detect the localization of NLRP3, a specific pyroptosis marker (Figure 3E). The mean fluorescence intensity (MFI) of NLRP3 in the RSF/DMF and RSF/DMF/ES groups was significantly lower than that in the RSF/DMSO and RSF/ES groups. Generally, these results confirmed that DMF/RSF/P:P conduits could inhibit SC pyroptosis on the cell surface. Subsequently, we examined the effects of culture supernatants from the five groups of Schwann cells on PC12 cell function by analyzing two neuronal structural proteins,^[^ ^48^ ^]^ neurofilament 200 (NF200) and the βIII tubulin isotype (Tuj1). The NF200 and Tuj1 levels in the RSF/DMF and RSF/DMF/ES groups were greater than those in the RSF/DMSO and RSF/ES groups, indicating that the DMF/RSF/P:P conduits with ES indirectly promoted PC12 cell function by inhibiting Schwann cell pyroptosis in vitro (Figure 3F,G). This result was confirmed using immunofluorescence staining (Figure 3H). ImageJ software was used to analyze the length and number of neurites of PC12 cells cultured with Schwann cell supernatants from the five groups. The results showed that the length and number of neurites in the RSF/DMF and RSF/DMF/ES groups were greater than those in the RSF/DMSO and RSF/ES groups (Figure S12, Supporting Information), suggesting that the combination of DMF/RSF/P:P conduits with ES indirectly promoted PC12 cell function.

#### 2.3.3 DMF/RSF/P:P Conduits Combined with ES Promote PC12 Cell Function by Inducing Macrophage Polarization Toward the M2 Subtype in Vitro

**Figure 4:**
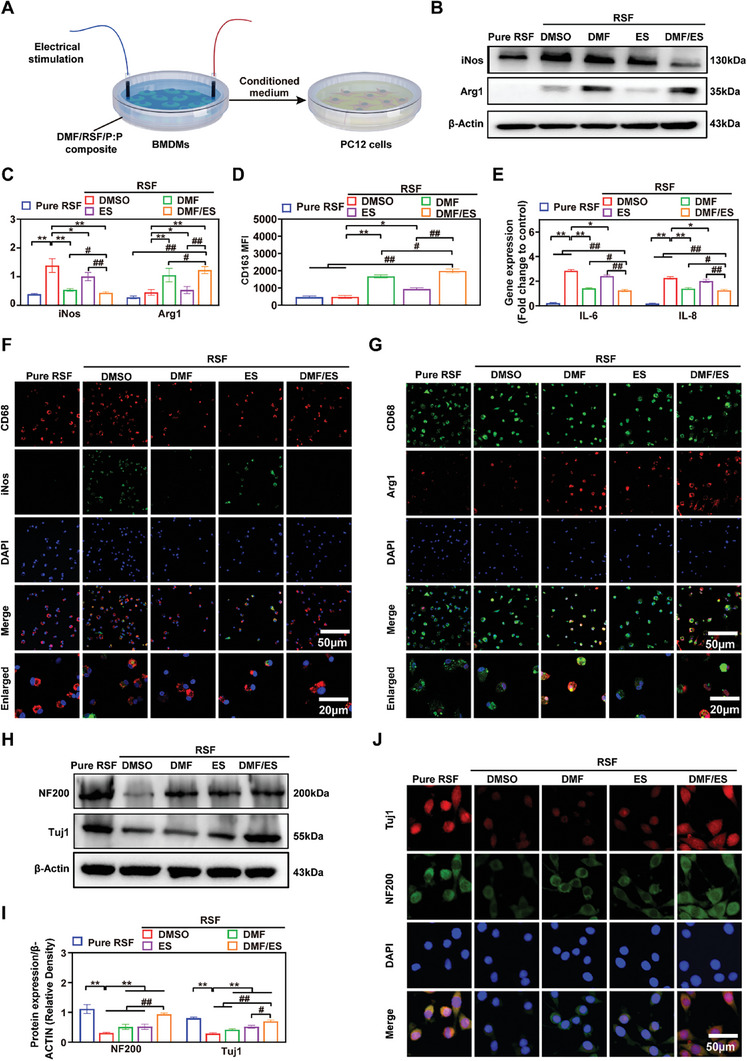
DMF/RSF/P:P conduits combined with ES promote PC12 cell function by inducing macrophage polarization toward the M2 subtype in vitro. A) Schematic diagram of DMF/RSF/P:P conduits combined with ES indirectly promoting PC12 cell function by facilitating M1 to M2 macrophage polarization. B,C) Protein levels and quantitative analysis of iNOS and Arg1 in BMDMs seeded in different conduit groups for 24 h. β‐Actin was used as an internal control. D) CD163 MFI in BMDMs treated with different interventions. E) Real‐time PCR analysis of IL‐6 and IL‐8 in BMDMs from different groups. F) Immunofluorescence staining for CD68 (red) and iNOS (green) in BMDMs treated with different interventions for 24 h. G) Immunofluorescence staining for CD68 (green) and Arg1 (red) in BMDMs from different groups. H,I) Protein levels and quantitative analysis of NF200 and Tuj1 in PC12 cells cultured in conditioned media from BMDMs treated with different interventions for 24 h. β‐Actin was used as an internal control. J) Immunofluorescence staining for NF200 (green) and Tuj1 (red) in PC12 cells cultured in conditioned media from BMDMs (DAPI: blue). Figure 4C–E,I were analyzed using one‐way ANOVA followed by Tukey's post hoc test and are presented as the means ± SDs. ^**^ *p* < 0.01 compared to the RSF/DMSO group. ^*^ *p* < 0.05 compared to the RSF/DMSO group. ^##^ *p* < 0.01 compared to the RSF/DMF/ES group. ^#^ *p* < 0.05 compared to the RSF/DMF/ES group. *n* = 3.

Previous studies reported that DMF treatment reduced the number of proinflammatory M1 macrophages in the rat sciatic nerve while increasing the number of anti‐inflammatory M2 macrophages,^[^ ^49^ ^]^ suggesting that DMF‐loaded DMF/RSF/P:P conduits combined with ES can induce macrophage polarization toward the M2 subtype and thereby remodel the IME. **Figure** **4**A shows a schematic diagram of DMF/RSF/P:P conduits combined with ES indirectly promoting PC12 cell function by facilitating M1 to M2 macrophage polarization. Bone marrow‐derived macrophages (BMDMs) seeded on the conduit material were divided into five groups (Pure RSF; RSF/DMSO, RSF/DMF, RSF/ES, and RSF/DMF/ES groups). Except for those in the Pure RSF group, the cells in all groups were induced by LPS/iFNr for 24 h. The protein level of nitric oxide synthase (iNOS) (M1) in the RSF/DMSO, RSF/ES, and RSF/DMF groups was significantly greater than that in the RSF/DMF/ES group. However, the protein level of Arg1 (M2) in the RSF/DMF/ES group was greater than that in the other four groups, indicating that both DMF/RSF/P:P conduits combined with ES could induce BMDMs on their surface to polarize toward the M2 subtype (Figure 4B,C). Flow cytometry analysis revealed that the CD163 (M2 subtype) MFI was significantly greater in the RSF/DMF/ES group than in the RSF/DMSO, RSF/ES, and RSF/DMF groups (Figure 4D). Moreover, real‐time polymerase chain reaction (RT–PCR) results suggested that inflammatory cytokine levels, including those of IL‐6 and IL‐8, were lower in the RSF/DMF/ES group than in the RSF/DMSO, RSF/ES, and RSF/DMF groups (Figure 4E). In addition, the trends in iNOS and arginase (Arg1) expression were similar according to the immunofluorescence staining results (Figure 4F,G). The results showed that DMF/RSF/P:P conduits combined with ES could effectively promote BMDM polarization toward the M2 subtype in vitro, and DMF played a major role in this process. We also cocultured PC12 cells with BMDM‐conditioned media to investigate the effect of BMDMs on PC12 cells. Western blot analysis and immunofluorescence staining revealed that treatment with BMDM‐conditioned media in the RSF/DMF/ES group enhanced PC12 cell function (Figure 4H‐J). Similarly, we analyzed the length and number of neurites in the five groups of PC12 cells cultured with BMDM supernatants (Figure S13, Supporting Information). The results showed that DMF/RSF/P:P conduits combined with ES could induce BMDM polarization toward the M2 subtype in vitro, indirectly promoting PC12 cell function.

### 2.4 Effects of Conductive DMF/RSF/P:P Conduits Combined with ES on Schwann Cell Pyroptosis and M2 Macrophage Polarization in Vivo

**Figure 5:**
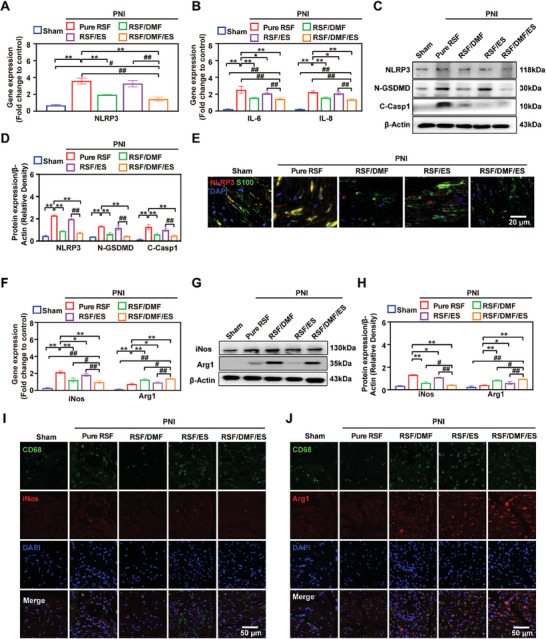
Effect of conductive DMF/RSF/P:P conduits on inhibiting Schwann cell pyroptosis and inducing macrophage polarization toward the M2 subtype in vivo. A,B) Real‐time PCR analysis of NLRP3 and inflammation‐related factors in regenerated nerve tissue. C,D) Protein levels and quantitative analysis of the pyroptosis‐related proteins NLRP3, N‐GSDMD, and C‐Casp1 in regenerated nerves 2 weeks after crush injury. β‐Actin was used as an internal control. E) Immunofluorescence staining for S100 (green) and NLRP3 (red) in regenerated nerves 2 weeks postoperatively (DAPI: blue). F) Real‐time PCR analysis of iNOS and Arg1. G,H) Protein levels and quantitative analysis of iNOS and Arg1 in the regenerated nerves. β‐Actin was used as an internal control. I,J) Immunofluorescence staining for CD68 (green) and Arg1 (red) in regenerated nerves 2 weeks postoperatively (DAPI: blue). Figure 5A,B,D,F,H were analyzed using one‐way ANOVA followed by Tukey's post hoc test and are presented as the means ± SDs. ^**^ *p* < 0.01 compared to the Pure RSF group. ^*^ *p* < 0.05 compared to the Pure RSF group. ^##^ *p* < 0.01 compared to the RSF/DMF/ES group. ^#^ *p* < 0.05 compared to the RSF/DMF/ES group. *n* = 6.

After PNI, Wallerian degeneration occurs in the distal region of the nerve.^[^ ^50^ ^]^ The main cellular agents controlling this process are Schwann cells and macrophages of the peripheral nervous system.^[^ ^51^ ^]^ Cell pyroptosis plays an important role in the process of peripheral nerve regeneration,^[^ ^4^, ^52^ ^]^ and a role for Schwann cell pyroptosis was confirmed in previous studies. When PNI occurs, pyroptosis of various cells in the microenvironment, particularly Schwann cells, can release several inflammatory factors into the microenvironment, impairing peripheral nerve regeneration.^[^ ^53^ ^]^ On the 7th postoperative day, we performed RT‒PCR analysis of sciatic nerve tissues from the five groups (sham, pure RSF, RSF/DMF, RSF/ES, and RSF/DMF/ES groups). Except for those in the sham group, the four groups were subjected to the same procedure to establish the peripheral nerve crush model.^[^ ^54^ ^]^ The RT‒PCR results showed that the gene expression of NLRP3 and the inflammation‐related factors IL‐6 and IL‐8 in the RSF/DMF/ES and RSF/DMF groups was significantly downregulated compared with that in the Pure RSF and RSF/ES groups, although the levels were slightly greater than those in the sham group (**Figure** **5**A,B). Subsequently, Western blot analysis and immunofluorescence staining analysis confirmed these results. The levels of the pyroptosis‐related proteins NLRP3, N‐GSDMD, and C‐Casp1 were significantly lower in the RSF/DMF/ES group than in the Pure RSF and RSF/ES groups; these findings are similar to those of the RSF/DMF group, indicating that DMF but not ES inhibits pyroptosis in vivo (Figure 5C,D). In addition, immunofluorescence staining was used to detect the localization of the specific Schwann cell marker S100 and the specific pyroptosis marker NLRP3 (Figure 5E). The MFI of NLRP3 in the RSF/DMF and RSF/DMF/ES groups was significantly lower than that in the Pure RSF and RSF/ES groups, indicating that DMF‐loaded DMF/RSF/P:P conduits could effectively inhibit Schwann cell pyroptosis by releasing DMF during the regeneration of the crushed sciatic nerve.

Macrophages are a type of plastic cell that can be polarized toward a spectrum of phenotypes under different stimuli,^[^ ^55^ ^]^ and the two ends of this spectrum are defined as proinflammatory M1‐ and anti‐inflammatory/tissue‐regenerative M2‐subtype macrophages.^[^ ^56^ ^]^ Strategies inducing M1 to M2 conversion have been demonstrated to promote neuronal regeneration through the use of inflammation‐related factors (e.g., nitric oxide and reactive oxygen species), which can result in neural damage, impaired neurogenesis, and particularly astrogliosis to form a glial scar to prevent axonal elongation.^[^ ^57^ ^]^ Two L‐arginine catalytic enzymes, iNOS and Arg1, are well‐characterized hallmark molecules of M1 and M2 macrophages, respectively.^[^ ^58^ ^]^ After PNI, large numbers of macrophages accumulate at the injury site, where they contribute to Wallerian degeneration and polarize to an anti‐inflammatory phenotype (M2) in the local microenvironment,^[^ ^59^ ^]^ promoting axonal regeneration.^[^ ^60^ ^]^ The expression of the Arg1 gene in the RSF/DMF/ES group was significantly greater than that in the other four groups, and the expression of the iNOS was inhibited in the RSF/DMF/ES group, indicating that the DMF/RSF/P:P conduits combined with ES effectively induced the polarization of macrophages into the M2 subtype in vivo (Figure 5F). Subsequently, Western blot analysis verified these results (Figure 5G,H). Furthermore, immunofluorescence staining was used to detect the localization of iNOS (Figure 5I) and Arg1 (Figure 5J). The MFI of Arg1 and iNOS in the RSF/DMF/ES group was significantly greater than that in the other four groups. The results suggested that DMF/RSF/P:P conduits combined with ES could induce macrophage polarization toward the M2 subtype in vivo, and ES and DMF work synergistically to facilitate this process.

### 2.5 Conductive DMF/RSF/P:P Conduits Combined with ES for in Vivo Peripheral Nerve Regeneration

#### 2.5.1 Functional Evaluation of Nerve Regeneration

**Figure 6:**
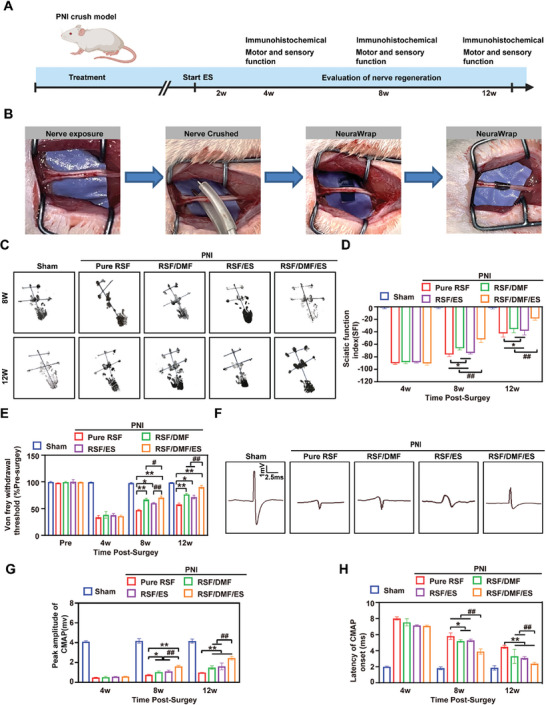
Functional assessment of nerve regeneration A) Schematic diagram of the peripheral nerve injury model and assessment of nerve regeneration. B) Repair of crushed nerves using DMF/RSF/P:P conduits. C,D) Footprints and gaits were recorded using SFI analysis at 4, 8, and 12 weeks postoperatively. E) Von Frey withdrawal threshold test (% preoperatively) at 4, 8, and 12 weeks postoperatively. F) Electrophysiological analysis. G) Peak amplitude of the CMAP at 4, 8, and 12 weeks postoperatively. H) Latency of CMAP onset in the five groups. Figure 6D,E,G,H were analyzed using one‐way ANOVA followed by Tukey's post hoc test and are presented as the means ± SDs. ^**^ *p* < 0.01 compared to the Pure RSF group. ^*^ *p* < 0.05 compared to the Pure RSF group. ^##^ *p* < 0.01 compared to the RSF/DMF/ES group. ^#^ *p* < 0.05 compared to the RSF/DMF/ES group; *n* = 6. ES, electrical stimulation; SFI, sciatic function index; CMAP, compound muscle action potential.

**Figure** **6**A shows a schematic diagram of the sciatic nerve crush injury model and treatment with DMF/RSF/P:P conduits combined with ES for 12 weeks. Figure 6B shows a schematic diagram of the animal surgery, which included nerve exposure, nerve crush, and nerve wrapping, and that the DMF/RSF/P:P conduits could be easily wrapped around the crushed nerve. SFI analysis was used to automatically record footprints and gaits in the five groups (sham, pure RSF, RSF/DMF, RSF/ES, and RSF/DMF/ES groups) at 4, 8, and 12 weeks postoperatively (Figure 6C,D). SD rats in the sham and RSF/DMF/ES groups showed better walking ability based on shorter footprints and distinguishable toes than did those in the Pure RSF group (Figure 6C). In addition, the SFI values showed that rats in the RSF/DMF/ES group had a faster recovery rate of motor function than did those in the Pure RSF, RSF/DMF, and RSF/ES groups at 8 and 12 weeks postoperatively, but no significant difference was observed at 4 weeks postoperatively (Figure 6D), possibly because nerve regeneration has not yet started at this stage, which was consistent with the findings of previous studies.^[^ ^61^ ^]^ The mechanical sensitivity test (Von Frey) consists of thin calibrated plastic filaments applied to the plantar surface of the hind paw.^[^ ^62^ ^]^ Von Frey filaments of varying thickness or stiffness were used to determine the threshold to elicit a hind‐withdrawal response.^[^ ^63^ ^]^ The von Frey test was used to evaluate the functional recovery of the crushed nerve at 4, 8, and 12 weeks postoperatively. No significant difference was observed in the 4th week, but the von Frey test showed that rats in the RSF/DMF/ES group had a faster rate of functional recovery than those in the Pure RSF, RSF/DMF, and RSF/ES groups at 8 and 12 weeks postoperatively (Figure 6E). Electrophysiology was used to further evaluate the functional recovery of the regenerated nerve and its affected muscles (Figure 6F). The compound muscle action potential (CMAP) peak amplitude was high in the regenerated nerve in the RSF/DMF/ES group (Figure 6G), as was the CMAP onset latency (Figure 6H), which was similar to that in the sham group.

#### 2.5.2 Remyelination of Regenerated Nerves

**Figure 7:**
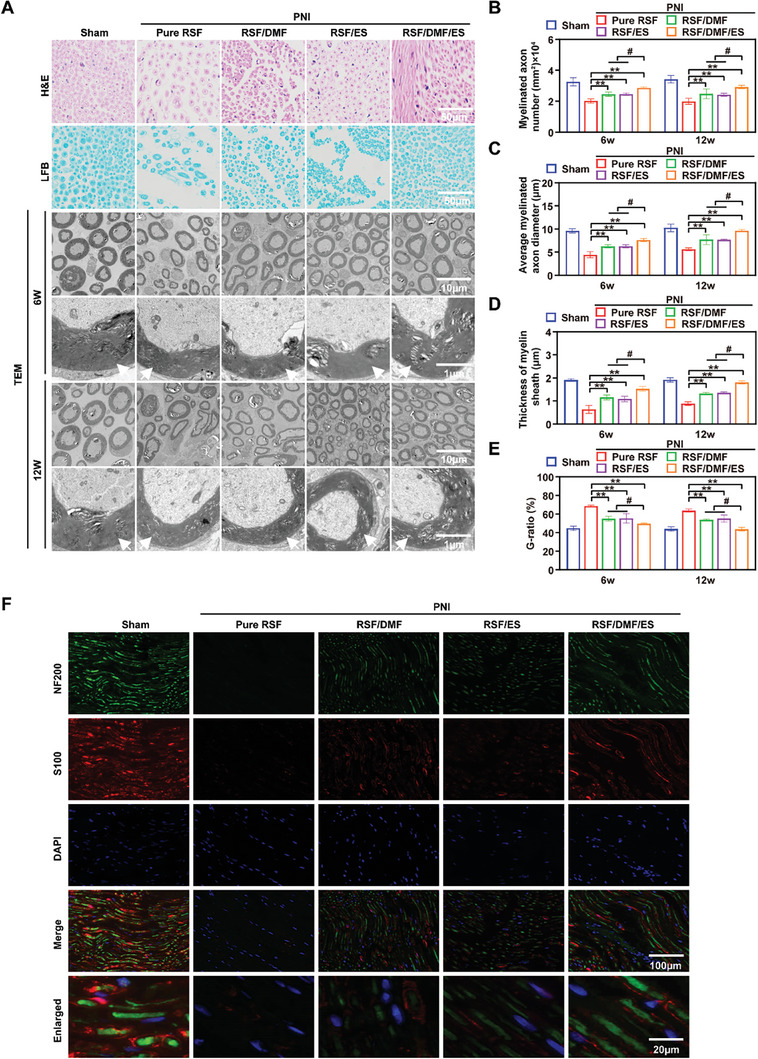
Remyelination of regenerated nerves. A) H&E staining, LFB staining, and TEM analysis of regenerated nerves. B) Several myelinated axons. C) Mean diameter of myelinated axons (µm). D) Myelin sheath thickness (µm). E) G‐ratio. F) Immunofluorescence staining for NF200 (green) and S100 (red) in the regenerated nerve at 12 weeks postoperatively (DAPI: blue). Figure 7B–E were analyzed using one‐way ANOVA followed by Tukey's post hoc test and are presented as the means ± SDs. ^**^ *p* < 0.01 compared to the Pure RSF group. ^*^ *p* < 0.05 compared to the Pure RSF group. ^##^ *p* < 0.01 compared to the RSF/DMF/ES group. ^#^ *p* < 0.05 compared to the RSF/DMF/ES group. *n* = 6 H&E, hematoxylin–eosin; LFB, Luxol fast blue; TEM, transmission electron microscopy. Red arrowheads indicate myelinated nerve fibers.

Hematoxylin–eosin (H&E) staining, Luxol fast blue (LFB) staining, and TEM were used to examine remyelination of the regenerated nerves (**Figure** **7**A). H&E staining revealed that the sizes of myelinated nerve fibers from rats in the sham and RSF/DMF/ES groups were similar. The myelin sheath appeared round, dense, and uniform, with an ordered lamellar structure showing neither axonal shrinkage nor axonal swelling. However, the myelin sheaths of the myelinated nerve fibers in the Pure RSF group were loose, thin, and disorganized. Compared with those in the Pure RSF, RSF/DMF, and RSF/ES groups, the LFB staining intensity in the regenerated nerves in the RSF/DMF/ES group was significantly greater during euthanasia. TEM revealed that the regenerated nerves in the RSF/DMF/ES group had more rich myelinated nerve fibers than those in the Pure RSF, RSF/DMF, and RSF/ES groups. The myelin sheaths in the RSF/DMF/ES and sham groups exhibited a typical stratified structure with a clear edge surrounding the entire crushed nerve fiber.^[^ ^9^ ^]^ Biostatistical analysis was used to quantitatively evaluate the degree of myelination of the regenerated nerve fibers, including the number of myelinated axons (Figure 7B), mean diameter of myelinated axons (Figure 7C), myelin sheath thickness (Figure 7D), and G‐ratio (%) (Figure 7E), at 6 and 12+6 weeks. The axons in the RSF/DMF/ES and sham groups underwent more myelination than those in the other three groups. Moreover, the axons in the RSF/DMF/ES group also exhibited significantly greater remyelination in terms of the mean myelinated axon diameter and myelin sheath thickness than did those in the Pure RSF, RSF/DMF, and RSF/ES groups, which were not significantly different from those in the sham group. In addition, the axons in the RSF/DMF/ES group also had a lower G ratio (%) than did those in the Pure RSF, RSF/DMF, and RSF/ES groups at 6 and 12 weeks postoperatively. The expression levels of the axon and Schwann cell biomarkers NF200 and S100 in the regenerated nerve were further evaluated (Figure 7F). Similarly to those in the sham group, NF200 and S100 expression in the RSF/DMF/ES group was significantly greater than that in the Pure RSF, RSF/DMF, and RSF/ES groups (Figure 7G). These data showed that the RSF/DMF/ES group had satisfactory recovery of nerve function, similar to the nerve function in the sham group.

#### 2.5.3 Reinnervated Gastrocnemius Muscle Examination

**Figure 8:**
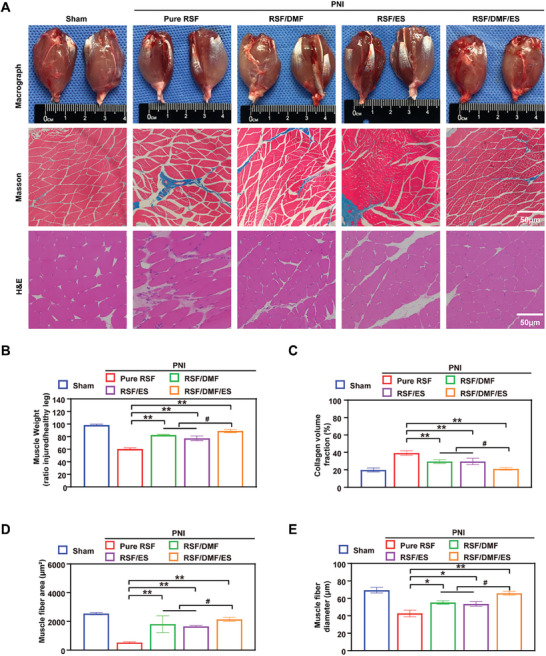
Examination of the reinnervated gastrocnemius muscle. A) Digital images, Masson, and H&E analysis of the gastrocnemius muscle. B–E) Muscle weight (ratio of injured/healthy leg), collagen volume fraction (%), muscle fiber area, and muscle fiber diameter were compared between groups. The data are expressed as the mean ± standard deviation. Figure 8B–E were analyzed using one‐way ANOVA followed by Tukey's post hoc test and are presented as the means ± SDs. ^**^ *p* < 0.01 compared to the Pure RSF group. ^*^ *p* < 0.05 compared to the Pure RSF group. ^##^ *p* < 0.01 compared to the RSF/DMF/ES group. ^#^ <*p* 0.05 compared to the RSF/DMF/ES group. *n* = 6 H&E, hematoxylin–eosin; LFB, Luxol fast blue; TEM, transmission electron microscopy.

Macrograph, Masson, and H&E analyses of the muscle were performed to evaluate the effect of our treatment approach on the organs of the mice (**Figure** **8**A). Muscle atrophy is a common debilitating symptom associated with PNI.^[^ ^64^ ^]^ We analyzed the gastrocnemius muscle, which is innervated by the tibial branch of the sciatic nerve. At 12 weeks postoperatively, the rats were euthanized, and their gastrocnemius muscles were collected to obtain the gastrocnemius muscle index by calculating the ratio of gastrocnemius muscle weight between the injured and healthy legs (Figure 8B). No significant differences were observed between the sham and RSF/DMF/ES groups, as determined by macrographing, Masson staining, or H&E staining of the muscle. The fiber area (Figure 8D) and diameter (Figure 8E) in the RSF/DMF/ES group were significantly greater than those in the Pure RSF, RSF/DMF, and RSF/ES groups at 12 weeks postoperatively, but the RSF/DMF/ES and sham groups showed no significant differences. Furthermore, the collagen volume fraction (%) in the sham and RSF/DMF/ES groups was significantly lower than that in the other three groups (Figure 8C). These results showed that DMF/RSF/P:P conduits with ES could alleviate muscle atrophy after crushed sciatic nerve denervation, indicating that ES can promote motor function recovery after PNI.

#### 2.5.4 In Vivo Biosafety of DMF/RSF/P:P Conduits

We evaluated the biosafety of DMF/RSF/P:P conduits in vivo. We further monitored tissue toxicity via H&E staining of major organs, including the heart, liver, spleen, lungs, and kidneys, indicating high histocompatibility of the composite conduits (Figure S14, Supporting Information). In addition, the levels of red blood cells (RBCs), white blood cells (WBCs), hemoglobin (HGB), alanine aminotransferase (ALT), aspartate aminotransferase (AST), creatinine (CRE), and blood urea nitrogen (BUN) were not significantly different between the Pure RSF and DMF/RSF/P:P groups (Figure S15, Supporting Information), indicating that the degradation of the composite conduit products in vivo did not cause any apparent systemic toxicity.

#### 2.5.5 The Effect of Electrical Stimulation on Degradability and Drug Release

We evaluated the effect of electrical stimulation on the degradability and drug release of DMF. The results indicate that electrical stimulation did not significantly impact the degradation performance of NGC (Figure S16A, Supporting Information), possibly because the effect of electrical stimulation on degradation is very slight compared to that of degrading enzymes and is therefore overshadowed by the degrading effect of the enzymes. Moreover, the results showed that the drug release rate of NGC accelerated in the presence of electrical stimulation (Figure S16B, Supporting Information), possibly because each electrical stimulation process caused the conductive polymer to undergo oxidation‐reduction reactions, leading to charge repulsion reactions and resulting in volume changes.^[^ ^65^ ^]^ This mechanical energy from the volume changes acts on the conductive film, ultimately causing the release of DMF, which is consistent with reports in related literature.^[^ ^66^ ^]^

## 3 Conclusions

Generally, we designed and developed novel multifunctional conductive DMF/RSF/P:P conduits based on RSF and bioencapsulated PEDOT:PSS, which can inhibit Schwann cell pyroptosis and induce macrophage polarization toward the M2 subtype to improve peripheral nerve regeneration. Our study showed a promising strategy to promote peripheral nerve regeneration by remodeling the intraconduit IME at the nerve injury site.

## 4 Experimental Section

Methods and associated references are available in the Supporting Information. All animal surgeries were performed according to the Use Guide of Experimental Animal Care of Fudan University. The experimental procedure of the sciatic nerve crush model was approved by the Ethics Committee of Zhongshan Hospital, Fudan University (approval number: Y2021‐228).

## Conflict of Interest

The authors declare no conflict of interest.

## Supporting information

Supporting Information

## Data Availability

The data that support the findings of this study are available from the corresponding author upon reasonable request.
