# Supplementary material for: Remodeling of the Intra‐Conduit Inflammatory Microenvironment to Improve Peripheral Nerve Regeneration with a Neuromechanical Matching Protein‐Based Conduit
Source: Adv Sci (Weinh). 2024 Mar 2;11(17):2302988. doi: 10.1002/advs.202302988 (PMC11077661; doi:10.1002/advs.202302988)
Supplement: Supplementary file 1 — Supporting Information [file ADVS-11-2302988-s001.pdf]

ADVANCED SCIENCE
Open Access

Supporting Information

for *Adv. Sci.*, DOI 10.1002/advs.202302988

Remodeling of the Intra-Conduit Inflammatory Microenvironment to Improve Peripheral Nerve Regeneration with a Neuromechanical Matching Protein-Based Conduit

Jia-Yi Wang, Ya Yuan, Shu-Yan Zhang, Shun-Yi Lu, Guan-Jie Han, Meng-Xuan Bian, Lei huang, De-Hua Meng, Di-Han Su, Lan Xiao, Yin Xiao, Jian Zhang, Ning-Ji Gong* and Li-Bo Jiang**

Supporting Information

Remodeling of the Intra-conduit Inflammatory Microenvironment to Improve Peripheral Nerve Regeneration with a Neuromechanical Matching Protein-based Conduit

Jia-Yi Wang[†1,] *Ya Yuan*[†1,2], *Shu-Yan Zhang*[†3], *Shun-Yi Lu*[† 1], *Guan-Jie Han*[1], *Meng-Xuan Bian*[1], *Lei huang*[1], *De-Hua Meng*[1], *Di-Han Su*[1], *Lan Xiao*[4,5], *Yin Xiao*[4,5,6], *Jian Zhang*[*1], *Ning-Ji Gong*[*7], *Li-Bo Jiang*[*1]

Affiliations

[1]Department of Orthopaedic Surgery, Zhongshan Hospital, Fudan University, Shanghai, 200032, China

[2]Department of Rehabilitation, Zhongshan Hospital, Fudan University, Shanghai, 200032, China

[3]The Key Laboratory for Ultrafine Materials of Ministry of Education, Engineering Research Centre for Biomedical Materials of Ministry of Education, Frontiers Science Center for Materiobiology and Dynamic Chemistry, School of Materials Science and Engineering, East China University of Science and Technology, Shanghai 200237, China

[4]School of Mechanical, Medical and Process Engineering, Centre for Biomedical Technologies, Queensland University of Technology, Brisbane, 4059, Australia

[5]Australia-China Centre for Tissue Engineering and Regenerative Medicine, Queensland University of Technology, Brisbane, 4059, Australia

[6]School of Medicine and Dentistry & Menzies Health Institute Queensland, Griffith University, Gold Coast 4222, Australia

[7]Department of Emergency, Department of Orthopedics, The Second Hospital, Cheeloo College of Medicine, Shandong University, Jinan, Shandong, 250033, China

[*] These are corresponding authors.

Email: Jian Zhang, zhang.jian@zs-hospital.sh.cn

Ning-Ji Gong, gongningji@sdu.edu.cn

Li-Bo Jiang, jiang.libo@zs-hospital.sh.cn

Tel: +86-021-64041990

[†]Jia-Yi Wang, Ya Yuan, Shu-Yan Zhang and Shun-Yi Lu contributed equally to this work.

Keywords: Peripheral Nerve Regeneration; Silk Fibroin; Electrical Stimulation; Neuromechanical Matching; Intra-conduit Inflammatory Microenvironment

Experimental Section

1.1 Fabrication of DMF/RSF/P:P conduits

We prepared the RSF aqueous solution following a previously described protocol.[1] Briefly, raw silk was boiled in a Na_2CO_3 solution (0.02 M) at 100 °C for 30 min, washed with distilled water and dried in a 37 °C incubator for 3 days. The extracted silk was dissolved in aqueous LiBr solution (9.3 mol/L) at 60 °C for 1 h. Subsequently, the as-prepared solution was dialyzed against deionized water by using a dialysis conduit (MWCO) for 3 days at room temperature. Finally, the RSF solution was concentrated with an aqueous polyethylene glycol (PEG) solution (molecular weight, 20 kDa) for 1 day.

We prepared a DMF/RSF/P:P composite solution to obtain a DMF/RSF/P:P conduit. Briefly, different volumes of a PEDOT:PSS aqueous solution (Lot: #739324, Sigma–Aldrich) and a DMF solution (Lot: #242926, Sigma–Aldrich) were added to different RSF concentrations (4, 8, and 12 wt%) to reach the final target concentration. Subsequently, the same volume of HRP (900 U ml^{-1}; Lot: #77332; Sigma–Aldrich) and H_2O_2 solution (0. 3% v/v) was added to the mixed solution at a ratio of 1:25 to promote cross-linking.[2] The solution was thoroughly mixed and poured into a flat-bottomed polystyrene well to dry into a conduit, which was dried overnight at room temperature and then soaked in an ethanol (75% *v/v*) solution for 6 h, making it insoluble in water. Finally, the DMF/RSF/P:P conduit was soaked in poly-L-lysine solution (0.1% w/v; lot: # P8920; Sigma–Aldrich) for 2 h to promote cell adhesion for subsequent *in vivo* and *in vitro* experiments.[3]

1.2 Material characterization

SEM (SEM, 400 Field Emission-SEM, USA) and Cryo-SEM (Glacios, ThermoFisher, Massachusetts, USA) were used to observe the microstructures of the DMF/RSF/P:P conduits. The samples were coated with a thin Au/Pd layer before analysis. We observed the fracture structure and surface morphology of the conduits containing different RSF concentrations (pure, 4 wt%, 8 wt%, and 12 wt%) with or without treatment with poly-L-lysine. A spectrometer (Nicolet iS20, Thermo Scientific, Waltham, MA, USA) was used to record the FT-IR spectra collected from 4000 to 400

cm^{-1}.

An atomic force microscope (Bruker Dimension ICON, Germany) was used for AFM analyses. X-ray diffraction (XRD, Ultima IV, Rigaku, Japan) was performed to analyze the crystalline structure of each sample. A mechanical tester (CMT6103, MTS, Minneapolis, USA) was used to examine the mechanical performance of the prepared conduits in the wet and dry states. Samples in the dry state were dried at room temperature for 48 h before testing. In contrast, the wet conduits were soaked in deionized water for 24 h before testing.[4] A digital multimeter (Keithley DMM-6500, Tektronix, Oregon, USA) with a 2-wire mode and a 100-ms resolution was used to evaluate the resistivity of the conduits with a gradient of PEDOT:PSS content (0.1%, 0.2%, and 0.3% w/v). A surface roughness tester (Surftest 402, Mitutoyo, Kanagawa, Japan) was used to measure the surface roughness. A JY-82B DSA Hydrophilicity System (Kruss, Hamburg, Germany) was used to evaluate hydrophilicity. Finally, a Bruker NanoStar system (Bruker AXS, Karlsruhe, Germany) was used to measure WAXS.

1.3 Degradation and DMF release of the DMF/RSF/P:P conduits *in vitro*

We prepared a protease XIV solution (0.1 U/mL; Lot: #P5147, Sigma–Aldrich) for degradation analysis.[3b] The wet weight of DMF/RSF/P:P conduits with a gradient of RSF content (4, 8, and 12 wt%) was measured initially and then after 7, 14, 21, and 28 days of incubation. The mass percent of the conduits was calculated using the following equation: mass percent = $W_t/W_0 \times 100\%$, where W_0 is the initial wet weight and W_t is the wet weight measured at different times after incubation.

HPLC was used to measure DMF release from DMF/RSF/P:P conduits.[5] Drug release was performed initially and after 7, 14, 21, and 28 days of incubation in a container with protease XIV solution. The conduits were incubated for different durations and subsequently crushed, followed by DMF extraction with ethanol.[6] The concentration of each sample was measured at baseline and after 7, 14, 21, and 28 days of incubation. The cumulative drug release (%) was calculated using the following

equation: cumulative drug release (%) = $(1 - R_t/R_0) \times 100\%$, where R_0 is the initial DMF content and R_t is the DMF content measured at different times after incubation. The measurement conditions were as follows: Ultimate Plus-C18 4.6 × 150 mm, 5 μm; UV measuring wavelength, 230 nm; mobile phase, water/methanol (60:40); flow rate, 1 mL/min; and sample injection volume, 5 μL.

1.4 Biocompatibility evaluation using cell culture *in vitro*

We used CCK-8 (Beyotime, Shanghai, China) and live/dead cell assays to evaluate SC viability. A CCK-8 assay of Schwann cells treated with different concentrations of PEDOT:PSS (0, 0.1%, 0.2%, and 0.3% w/v) was performed in 96-well plates for 1, 2, and 3 days following the manufacturer's protocol. A microplate reader (Leica Microsystems, Wetzlar, Germany) was used to measure optical density at 450 nm. Moreover, a live/dead assay of Schwann cells treated with different concentrations of PEDOT:PSS for 1, 3, and 7 days was also performed using a working solution containing calcein-propionic acid (PI) and AM in 12-well plates. To determine the optimal DMF concentration, a CCK-8 assay was performed on Schwann cells seeded on DMF/RSF/P:P conduits with different DMF concentrations (0, 100, 200, 300, 400, and 500 μM) for 12, 24, and 48 h in 96-well plates.

1.5 Real-time PCR

We collected total RNA using TRIzol reagent (Lot: #R0016, Beyotime) and obtained cDNA after reverse transcription by using a Prime Script RT Reagent Kit (Lot: #RR047A, TaKaRa, Japan). The DESeq R package (2012) was used for differential expression analysis. Table S1 shows the PCR sequences of primers used**.**

1.6 ELISA

The culture supernatant of treated Schwann cells was harvested, and IL-1β (Lot: #E-EL-R0012c; Elabscience, China) and IL-18 (Lot: #E-EL-R0567c; Elabscience, China) levels were detected using ELISA kits following the manufacturer's protocols.

1.7 Western blot analysis

After routine protein extraction, electrophoresis, and membrane transfer, the membrane was blocked with skim milk (Lot: #232100, BD) for 1.5 h. Subsequently, the membrane was incubated overnight at 4 °C with the following diluted primary antibodies: β-actin antibody (Lot: #4967, CST), caspase-1 (Lot: #ab179515, Abcam), NLRP3 (Lot: #ab263899, Abcam), GSDMD (Lot: #E9S1X, CST), iNOS (Lot: #P35228, Abmart), Arg1 (Lot: #P05089, Abmart), NF200 (Lot: #GB11141, Servicebio), and Tuj1 (Lot: #GB11139, Servicebio). Finally, the sections were incubated with secondary antibodies for 1 h at room temperature.

1.8 Flow cytometry assay

BMDMs (1×10^5 cells/well) were seeded in 6-well plates and subjected to different interventions (pure RSF, RSF/DMSO, RSF/DMF, RSF/ES, and RSF/DMF/ES) for 24 h. Subsequently, adherent cells were separated by digestion with Accutase (Lot: #40506ES60, Yeasen) and collected for flow cytometry analysis with antibodies against CD68 (Lot: #MA528262, Thermo), CD86 (Lot: #746002, BD Pharmingen), CD11b (Lot: #562102, BD Pharmingen), and CD163 (Lot: #NB110–40686PE, Novus).

1.9 Rat PNI model and electrical muscle stimulation

Male SD rats aged 6–8 weeks were housed in temperature- and humidity-controlled rooms at the Laboratory Animal Center of Zhongshan Hospital, Fudan University. The rats were anesthetized, and their left hind leg was prepared for surgery after shaving and disinfection. The sciatic nerve was isolated and crushed following a previously described protocol.[7] In the sham group, the sciatic nerve was exposed without damage, and the muscle layers and skin were subsequently sutured. The sciatic nerve samples 1 cm distal to the nerve crush site were harvested in the PNI group, and nerve samples of the same length were also harvested in the sham group. The same operator performed all the procedures. In the study, the pyroptosis phenomenon was evaluated separately in the sciatic nerve crush model and the defect model, and the results showed that the pyroptosis appeared to be more pronounced in the crush model (Figure S17A, B,

Supporting Information). Before implantation, the “sheet” was soaked in PBS solution to soften it, making it easy to roll it up into a conduit. Then, the films were wrapped around the sciatic nerve and fixed to the nerve epineurium with 10-0 monofilament nylon sutures (Figure S17C, Supporting Information).[8] Then, the incisions in the skin and muscles were closed, and the subsequent experiments were performed. The animals in the ES group received ES daily for 2 weeks postoperatively using *in vivo* stimulation parameters (7 mA for 30 min). In brief, rats were placed in a fixation device (GEGDR1, Beijing Global Biotechnology Co., Ltd., China) with both hind limbs exposed as mentioned previously,[8] and two somatic electrodes were placed on the skin over the gastrocnemius muscle of the left leg and connected to a stimulator (XCH-B2, Shanghai NCC Electronics Co., Ltd., China) (on time = 5 s, off time = 10 s, pulse width = 200 μm).[9] The optimal current parameters for electrical stimulation were based on previous reports and preliminary experiments by the research group; that is, the lowest current value that can lead to visible toe and foot movements during electrical stimulation without causing significant pain or discomfort in the rats.[8, 10]

1.10 Culture of Schwann cells

Schwann cells were cultured in Dulbecco’s modified Eagle’s medium (DMEM, Gibco) supplemented with 10% fetal bovine serum (FBS, BI) and 1% antibiotics (100 μg/ml penicillin and 100 μg/ml streptomycin). In the ES groups, Schwann cells were treated with a custom-made ES device (Figure S18A, Supporting Information) according to previous methods.[11] Specifically, we drilled holes in the lids of six-well plates so that the needle of the syringe could be inserted vertically through the holes into the medium in the well plates. The exposed metal needle outside the well plate was connected to the electrical stimulation device by a wire. The determination of experimental parameters is based on previous research reports and the accumulation of experimental data by our research group (electrical frequency = 20 Hz, voltage intensity = 10 mV DC, and ES time of 1 h d^{-1}).[8] We evaluated cell compatibility under different electrical stimulation parameters and finally determined the most suitable parameters (10 mV DC, Figure S18B, Supporting Information).

1.11 Extraction and culture of primary BMDMs

Purified primary BMDMs were obtained from the bone marrow of male SD rats following a previous protocol.[12] Briefly, single-cell suspensions of bone marrow cells from the tibia and femur were prepared. The bone marrow cells were differentiated for 7 days in DMEM/F12 supplemented with 10% FBS and 50 ng/mL recombinant macrophage colony-stimulating factor (Novoprotein, China).

1.12 Coculture system

Conditioned medium was collected from Schwann cells and BMDMs and added to PC12 cells to assess cell survival and growth.[13] Subsequently, PC12 cell growth was observed after 24 h.

1.13 Functional evaluation of nerve regeneration

SFI analysis was performed at 4, 8, and 12 weeks postoperatively as previously described.[14] Briefly, the hind paws were dipped in black ink, and the rats were allowed to walk down a narrow channel, making footprints on white paper. The SFI was calculated as follows: SFI = −38.3 × (EPL − NPL)/NPL + 109.5 × (ETS−NTS)/NTS + 13.3 × (EIT−NIT)/NIT − 8.8. PL, TS, and IT are the distances from the heel to the third toe, from the first to fifth toes, and from the second to fourth toes, respectively. E and N represent the experimental and normal groups, respectively. To measure the paw withdrawal threshold, the von Frey test was performed for the experimental and control groups at 4, 8, and 12 weeks postoperatively as previously described.[15] Electrophysiological analysis was performed at 4, 8, and 12 weeks postoperatively to collect electrical signals from different experimental groups following a previous protocol.[16] Briefly, the left sciatic nerves of the rats were isolated and exposed after anesthesia. The stimulating electrodes were inserted into the proximal side of the sciatic nerve before wrapping the conductive conduits around the crushed peripheral nerve. Subsequently, the electrodes were connected to an external stimulator (MD3000-C, ZhengHua). The stimuli were delivered in pulses (stimulation intensity = 1 mA; duration = 0.2 ms; frequency = 1 Hz). Additionally, a signal-receiving electrode was placed on the outer paw of the stimulated

leg. Moreover, the distance between the stimulating and recording electrodes was measured. The peak amplitude of the CMAP and the latency to CMAP onset were calculated based on a previously published method.[17]

1.14 Immunofluorescence analysis

After the rats were anesthetized, sciatic nerve segments 1 cm distal to the nerve crush site were removed and fixed in mounting medium. Subsequently, the nerve sections were permeabilized, washed, and blocked in 5% bovine serum albumin for 1 h. The sections were incubated overnight at 4 °C with the following primary antibodies: S100 (Lot: #GB11359, Servicebio), NLRP3 (Lot: #GB114320, Servicebio), iNOS (Lot: #P35228, Abmart), Arg1 (Lot: #P05089, Abmart), and CD68 (Lot: #GB113109, Servicebio). The slides were incubated with secondary antibodies after three washes. A fluorescence microscope (Nikon, Tokyo, Japan) was used to visualize and capture the images.

Similarly, the cells were incubated *in vitro* with the following primary antibodies: NLRP3 (Lot: #T55651, Abmart), iNOS (Lot: #P35228, Abmart), Arg1 (Lot: #P05089, Abmart), CD68 (Lot: #GB113109, Servicebio), NF200 (Lot: #GB11141, Servicebio), and Tuj1 (Lot: #GB11139, Servicebio).

1.15 Histological evaluation and TEM analysis of regenerated nerves

The nerve segments 1 cm distal to the site of nerve crush in the rats were dissected and harvested at 12 weeks postoperatively. The nerve segments were cut into 8-μm-thick cross-sections (CM^3050S, Leica), stained with H&E and subjected to LFB analysis. Images from five random fields of each section were analyzed, and three samples from each group were statistically analyzed. The nerve segments 1 cm distal to the nerve crush site were harvested at 6 and 12 weeks postoperatively. Subsequently, the tissue samples were immersed in electron microscopy fixative and analyzed using TEM (JEOL, Tokyo, Japan). ImageJ software (NIH Image, Bethesda, MD) was used to evaluate the number of myelinated axons, mean diameter of myelinated axons, myelin sheath thickness, and G ratio (%) from the electron micrographs.[18]

1.16 Morphometric examination of the reinnervated muscles

The bilateral gastrocnemius muscles from each group were harvested at 12 weeks postoperatively. Afterward, the bilateral wet muscle weight was measured. The muscle samples were stained with H&E and Masson's trichrome. Images from five random fields of each sample were analyzed, and three samples from each group were statistically analyzed. ImageJ software (NIH, Bethesda, MD, USA) was used to measure the collagen volume fraction, muscle fiber area, and muscle fiber diameter from H&E-stained muscle section images.

1.17 Quantification and Statistical analyses

The data are expressed as the mean ± standard deviation (S.D.). GraphPad Prism version 8.0 (GraphPad Software, San Diego, CA, USA) was used for statistical analyses. One-way analysis of variance and Tukey's post hoc test were used to evaluate differences between groups ($n \geqslant 3$). For the Western blot and real-time PCR results, final normalization was achieved by calculating the ratio of the measured data to the reference data, followed by statistical analysis, while the remaining data were statistically analyzed directly from the measurement results. The significance of differences is reported as $^{**}p < 0.01$, $^{*}p < 0.05$, $^{\#\#}p < 0.01$, and $^{\#}p < 0.05$. $p < 0.05$ was considered to indicate statistical significance. Groups with no significant difference are not indicated by the symbol.

References

[1] M. Wu, Z. Han, W. Liu, J. Yao, B. Zhao, Z. Shao, X. Chen, *J Mater Chem B* **2021**, 9, 2025.

[2] Y. Liu, S. Ling, S. Wang, X. Chen, Z. Shao, *Biomater Sci* **2014**, 2, 1338.

[3] a)K. Cramer, A. L. Bolender, I. Stockmar, R. Jungmann, R. Kasper, J. Y. Shin, *Int J Mol Sci* **2019**, 20; b)A. Zhuang, X. Huang, S. Fan, X. Yao, B. Zhu, Y. Zhang, *ACS Appl Mater Interfaces* **2022**, 14, 123.

[4] D. Gunapu, Y. B. Prasad, V. S. Mudigunda, P. Yasam, A. K. Rengan, R. Korla, S. R. K. Vanjari, *Int J Biol Macromol* **2021**, 176, 498.

[5] Z. G. Ma, R. Ma, X. L. Xiao, Y. H. Zhang, X. Z. Zhang, N. Hu, J. L. Gao, Y. F. Zheng, D. L. Dong, Z. J. Sun, *Acta Biomater* **2016**, 44, 323.

[6] F. Ferrara, M. Benedusi, F. Cervellati, M. Sguizzato, L. Montesi, A. Bondi, M. Drechsler, W. Pula, G. Valacchi, E. Esposito, *Int J Mol Sci* **2022**, 23.

[7] A. L. Kalinski, C. Yoon, L. D. Huffman, P. C. Duncker, R. Kohen, R. Passino, H. Hafner, C. Johnson, R. Kawaguchi, K. S. Carbajal, J. S. Jara, E. Hollis, D. H. Geschwind, B. M. Segal, R. J. Giger, *Elife* **2020**, 9.

[8] S. Lu, W. Chen, J. Wang, Z. Guo, L. Xiao, L. Wei, J. Yu, Y. Yuan, W. Chen, M. Bian, L. Huang, Y. Liu, J. Zhang, Y. L. Li, L. B. Jiang, *Small Methods* **2023**, 7, e2200883.

[9] F. F. Tu, S. As-Sanie, J. F. Steege, *Obstet Gynecol Surv* **2005**, 60, 379.

[10] a)M. P. Willand, C. D. Chiang, J. J. Zhang, S. W. Kemp, G. H. Borschel, T. Gordon, *Neurorehabil Neural Repair* **2015**, 29, 690; b)T. Fu, L. Jiang, Y. Peng, Z. Li, S. Liu, J. Lu, F. Zhang, J. Zhang, *Neuroscience* **2020**, 426, 179.

[11] Z. Huang, M. Sun, Y. Li, Z. Guo, H. Li, *J Mater Chem B* **2021**, 9, 2656.

[12] A. Al Mamun, Y. Wu, I. Monalisa, C. Jia, K. Zhou, F. Munir, J. Xiao, *J Adv Res* **2021**, 28, 97.

[13] E. Pompili, C. Fabrizi, F. Somma, V. Correani, B. Maras, M. E. Schinina, V. Ciraci, M. Artico, F. Fornai, L. Fumagalli, *Mol Cell Neurosci* **2017**, 79, 23.

[14] Q. Cheng, C. Jiang, C. Wang, S. Yu, Q. Zhang, X. Gu, F. Ding, *Neural Regen Res* **2014**, 9, 2142.

[15] a)Y. Seto, F. Okazaki, K. Horikawa, J. Zhang, H. Sasaki, H. To, *BMC Cancer* **2016**, 16, 756; b)L. Singh, A. Kaur, S. Garg, A. P. Singh, R. Bhatti, *Neurochem Res* **2020**, 45, 2364.

[16] S. Song, K. W. McConnell, D. Amores, A. Levinson, H. Vogel, M. Quarta, T. A. Rando, P. M. George, *Biomaterials* **2021**, 275, 120982.

[17] S. Wang, C. Zhu, B. Zhang, J. Hu, J. Xu, C. Xue, S. Bao, X. Gu, F. Ding, Y. Yang, X. Gu, Y. Gu, *Biomaterials* **2022**, 280, 121251.

[18] L. Huang, X. Quan, Z. Liu, T. Ma, Y. Wu, J. Ge, S. Zhu, Y. Yang, L. Liu, Z. Sun, J. Huang, Z. Luo, *Tissue Eng Part A* **2015**, 21, 1409.

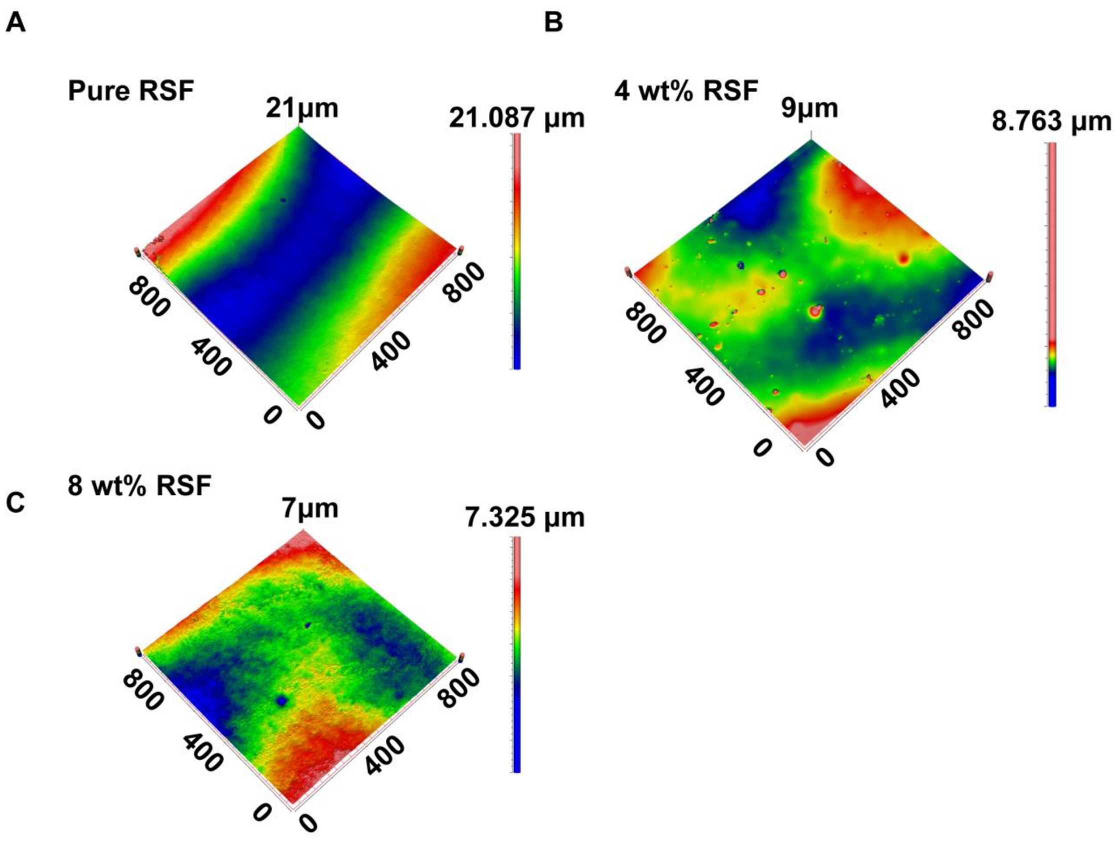

Figure S1. Surface roughness of the different conduit samples. (A) Control group. (B) DMF/4 wt% RSF/P:P group. (C) DMF/8 wt% RSF/P:P group. n = 3.

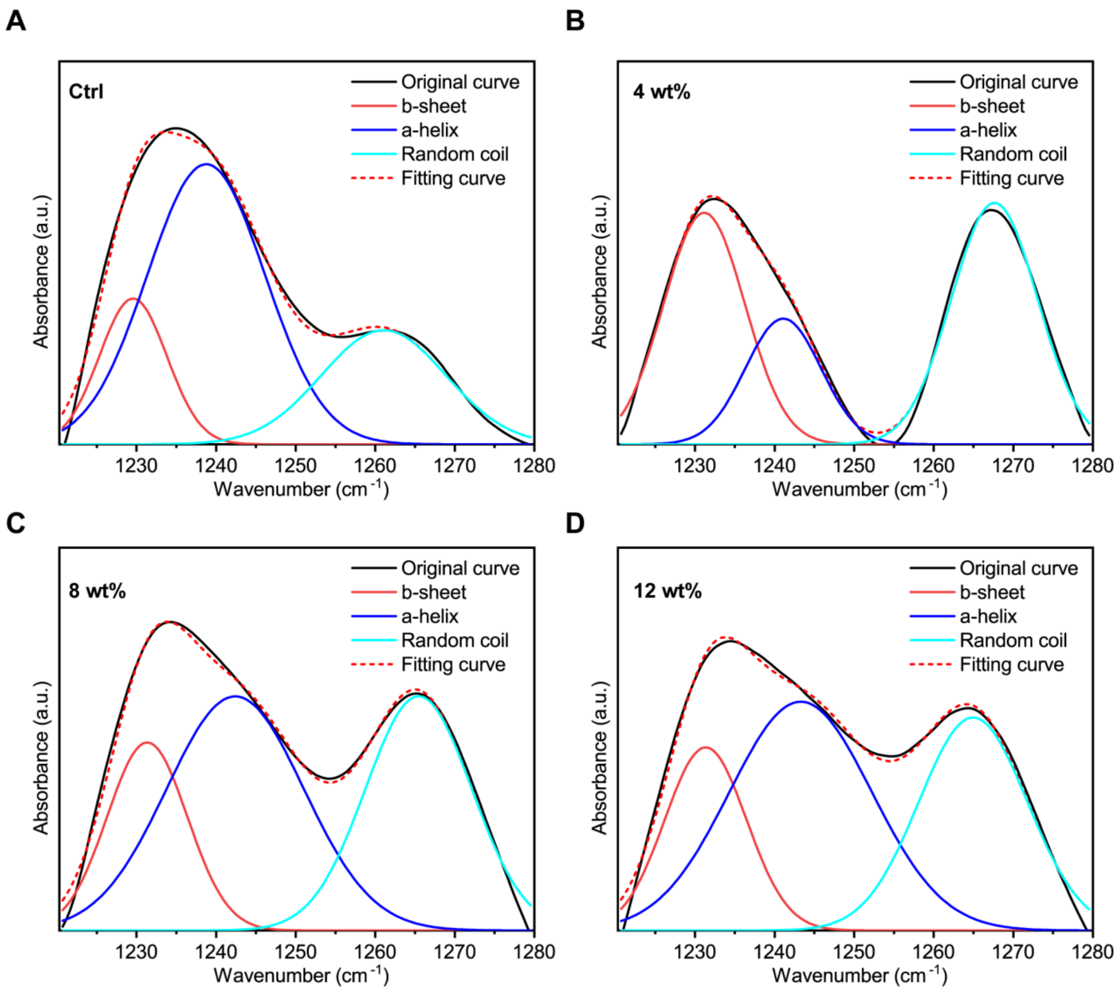

Figure S2. Fitting analysis of different conduits based on the amide I band in the FT-IR spectra. (A) Control group. B) DMF/4 wt% RSF/P:P group. (C) DMF/8 wt% RSF/P:P group. (D) DMF/12 wt% RSF/P:P group. n = 3.

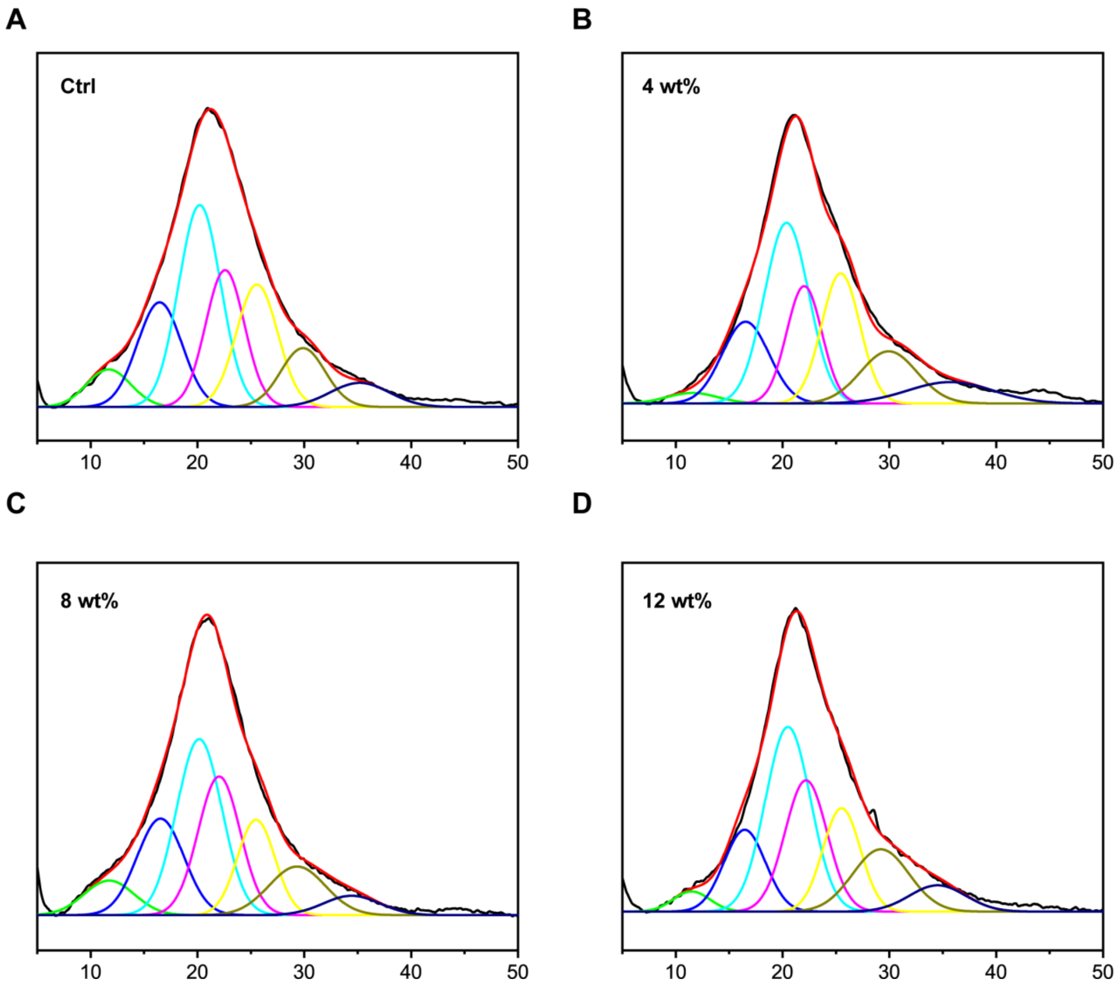

Figure S3. Fitting analysis of the XRD curves for each group. (A) Control group. (B) DMF/4 wt% RSF/P:P group. (C) DMF/8 wt% RSF/P:P group. (D) DMF/12 wt% RSF/P:P group. n = 3.

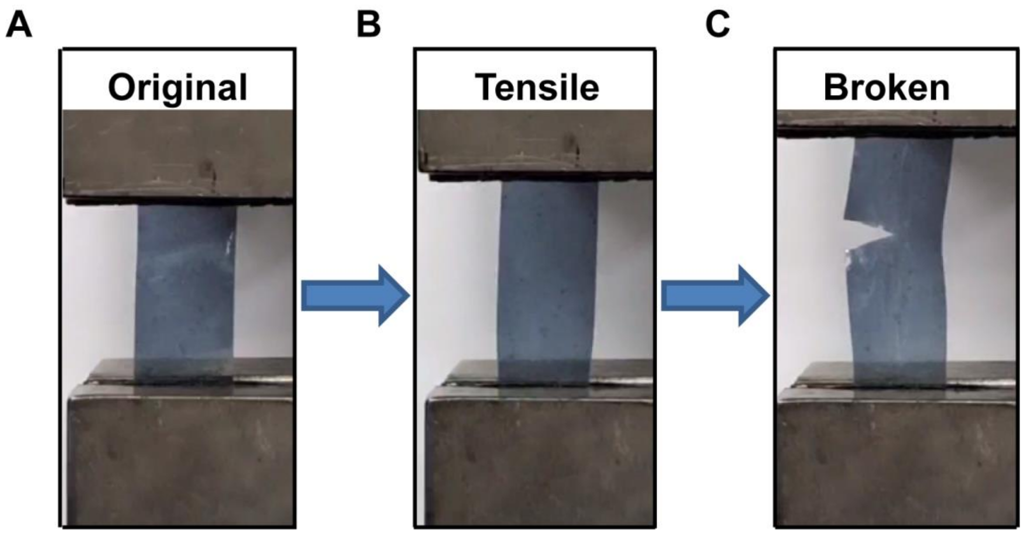

Figure S4. Digital photographs of DMF/RSF/P:P composites in a tensile test.

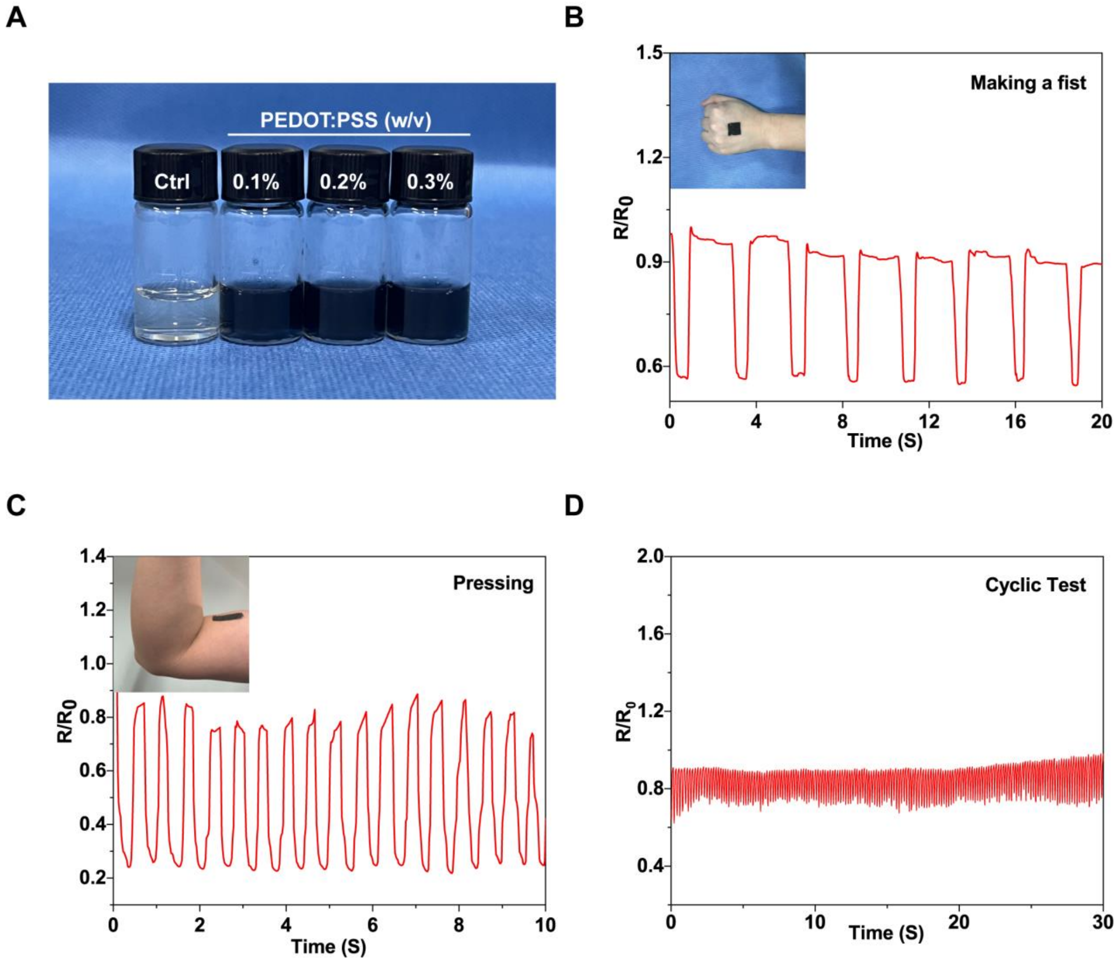

Figure S5. Multifunctional properties of DMF/RSF/P:P conduits. (A) Digital photos of preconduit solutions containing different concentrations of the conductive component PEDOT:PSS. (B–C) Demonstration of the sensor in tracking human movement. Sensors assembled from DMF/RSF/P:P conduits monitor various human movements. (B) Making a fist. (C) Pressing. (D) Cyclic test of DMF/RSF/P:P composites.

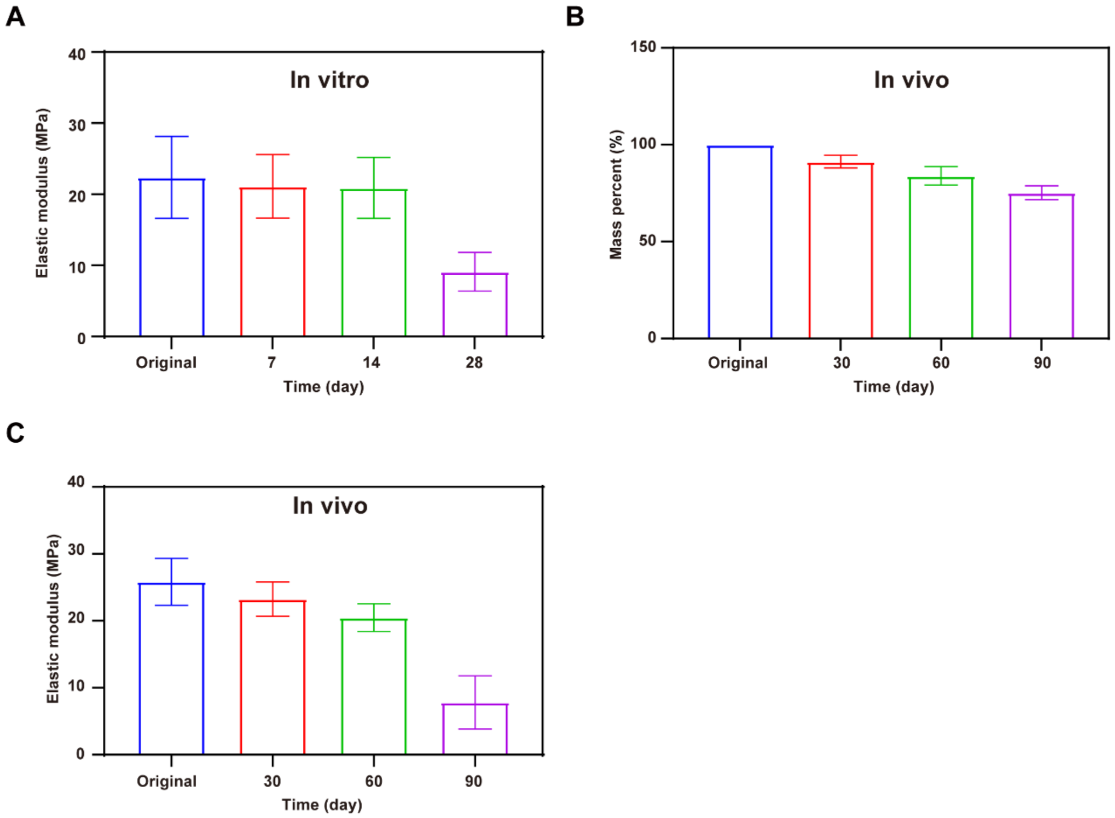

Figure S6. Degradation and mechanical performance of DMF/4 wt% RSF/P:P conduits *in vitro* and *in vivo*. (A) The elastic modulus of DMF/4 wt% RSF/P:P conduits at different time points *in vitro*. (B) Degradation behavior of DMF/RSF/P:P composites *in vivo*. (C) The elastic modulus of DMF/4 wt% RSF/P:P conduits at different time points *in vivo*. n = 3.

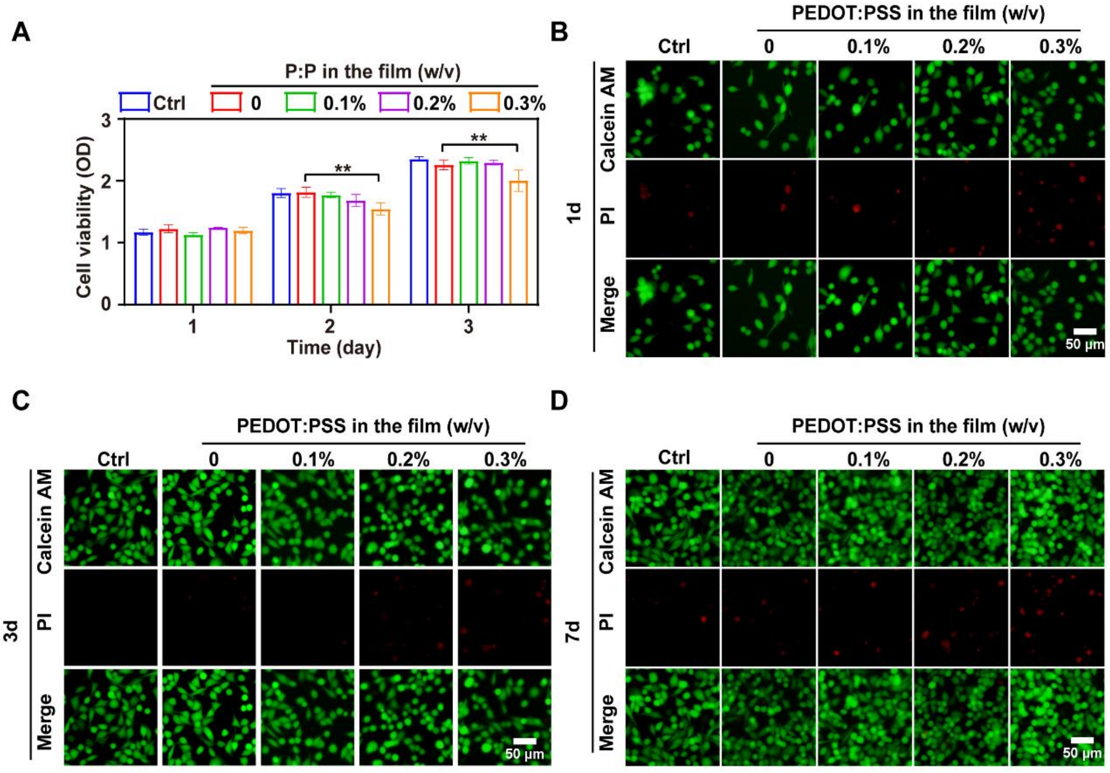

Figure S7. Identification of the optimum PEDOT:PSS concentration. (A) CCK-8 assay. (B–D) Live/dead staining on different conduits with varying PEDOT:PSS concentrations at 1, 3, and 7 days. All the data were analyzed using one-way ANOVA followed by Tukey's post hoc test and are presented as the means ± SDs. $**p < 0.01$, $*p < 0.05$, and ** indicate statistical significance between the indicated groups; n = 3.

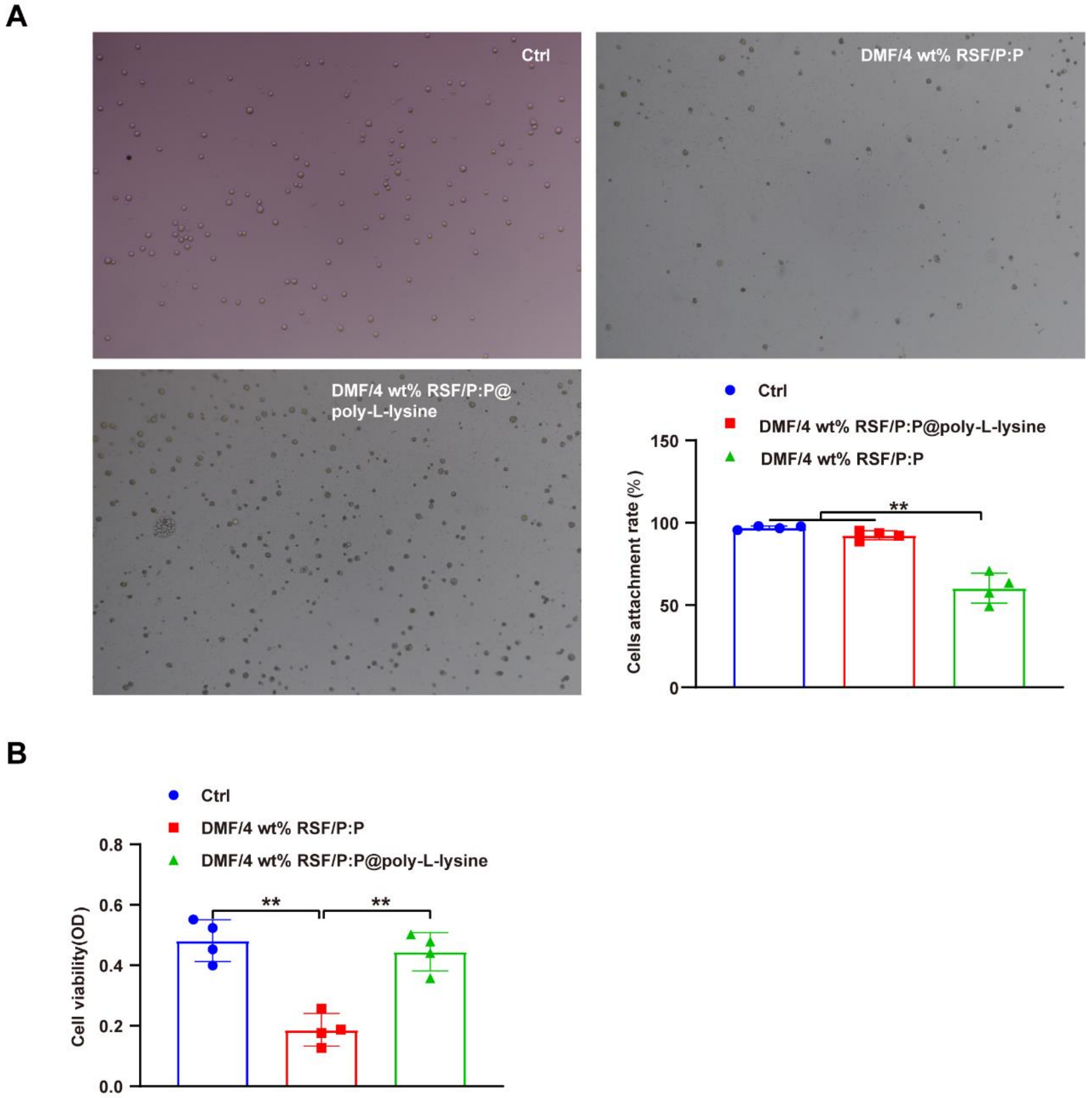

Figure S8. The cell adhesion properties of the DMF/RSF/P:P conduits in the presence or absence of poly-L-lysine solution treatment. (A) Cell adhesion properties of DMF/RSF/P:P conduits after 4 hours of Schwann cell plating *in vitro*. (B) The CCK-8 results after 4 hours of cell plating. All the data were analyzed using one-way ANOVA followed by Tukey's post hoc test and are presented as the means ± SDs. ***p* < 0.01, **p* < 0.05, and * and ** indicate statistical significance between the indicated groups. n = 3.

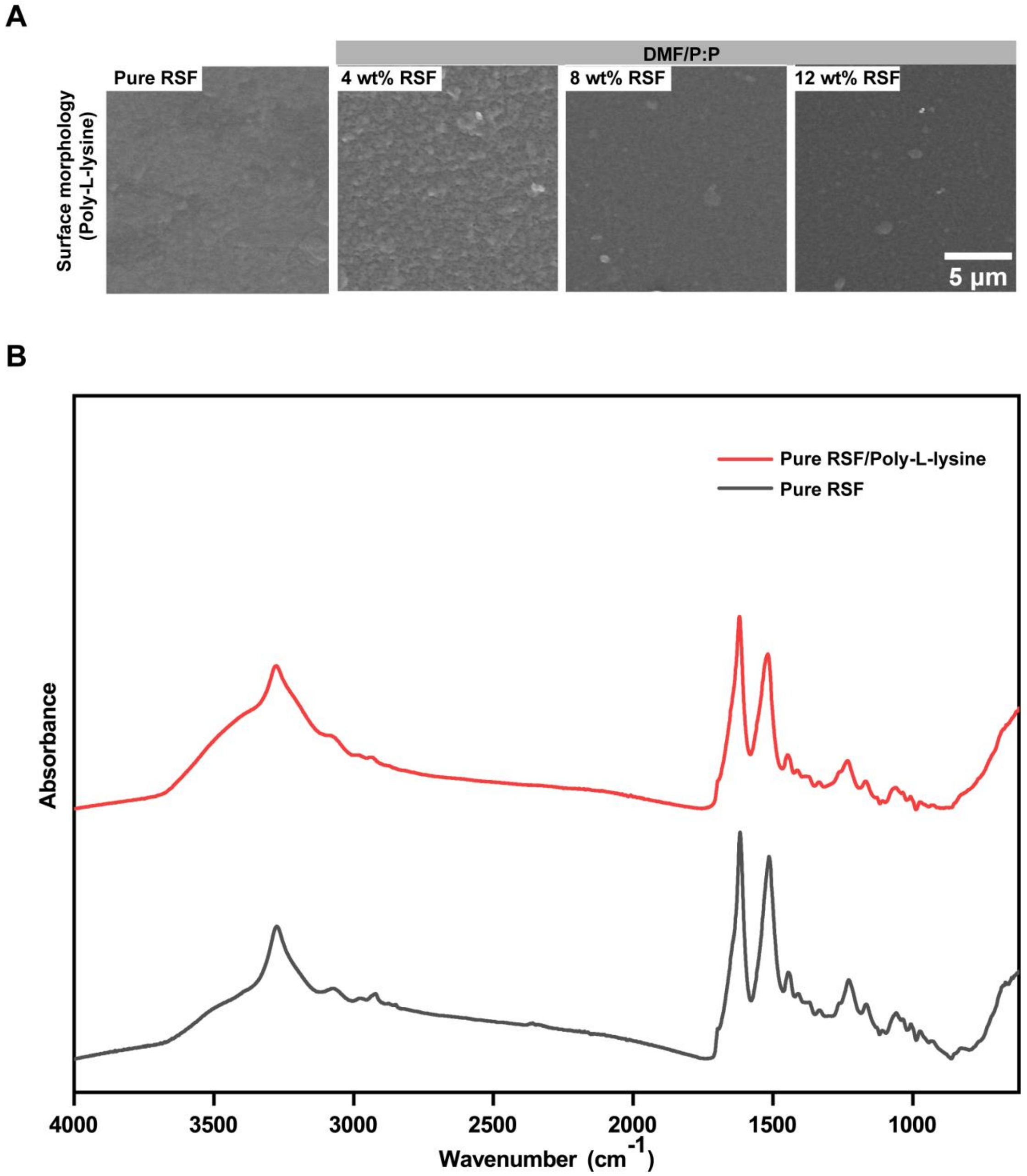

Figure S9. DMF/RSF/P:P composites treated with poly-L-lysine**.** (A) SEM images of the surface of the DMF/RSF/P:P conduits at 4, 8, and 12 wt% RSF concentrations with poly-L-lysine. (B) FT-IR results of the DMF/RSF/P:P composites in the presence or absence of poly-L-lysine. n = 3. SEM, scanning electron microscopy; FT-IR, Fourier transform infrared spectroscopy.

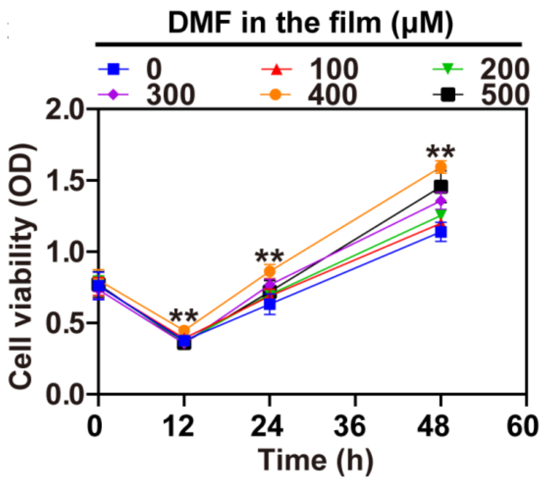

Figure S10. A CCK-8 assay was used to optimize the concentration of DMF loaded with DMF/RSF/P:P conduits. All the data were analyzed using one-way ANOVA followed by Tukey's post hoc test and are presented as the means ± SDs. $^{**}p < 0.01$, $^{*}p < 0.05$, and * and ** indicate statistical significance between the indicated groups. n = 3.

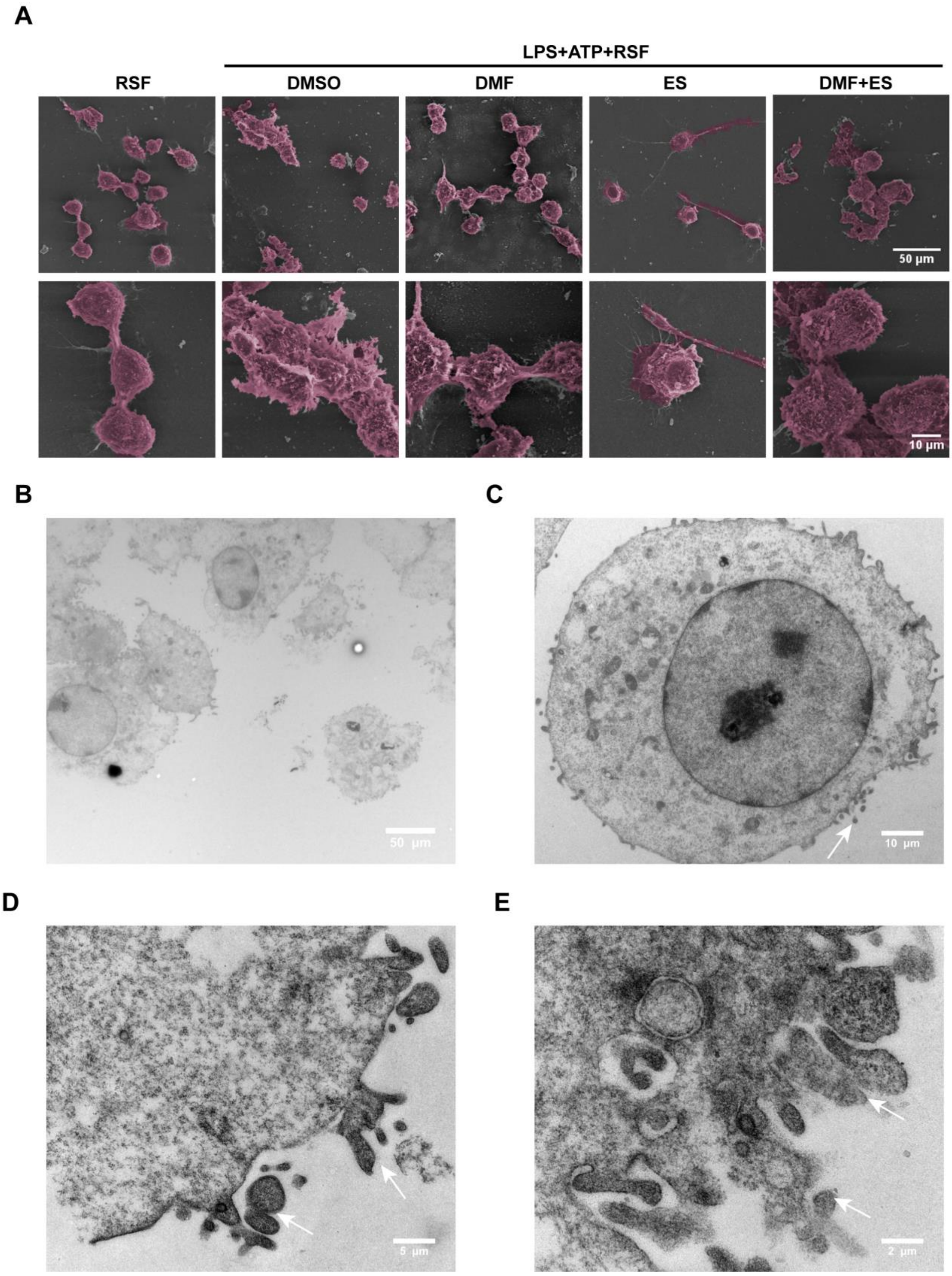

Figure S11. SEM and TEM images of Schwann cells in each group after treatment with LPS+ATP. (A). Representative SEM images of Schwann cells from the different groups are shown. (B–E) Representative TEM images of pyroptotic Schwann cells. The white arrowheads indicate large bubbles emerging from the cytoplasm of Schwann cells. SEM, scanning electron microscopy; FT-IR, Fourier transform infrared spectroscopy.

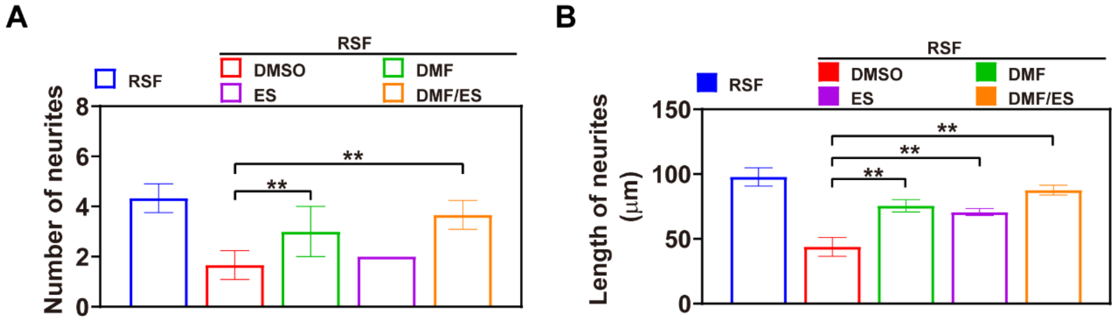

Figure S12. Functional assessment of PC12 cells after conditional coculture with Schwann cells. (A) Number and (B) length of neurites of PC12 cells cultured with Schwann cell supernatants in the five groups. All the data were analyzed using one-way ANOVA followed by Tukey's post hoc test and are presented as the means ± SDs. $^{**}p < 0.01$, $^{*}p < 0.05$, and ** indicate statistical significance between the indicated groups; n = 6.

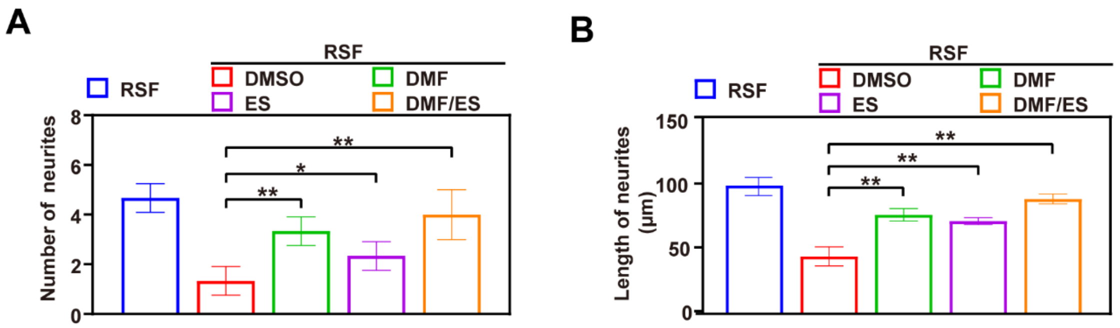

Figure S13. Functional assessment of PC12 cells after conditional coculture with BMDMs. (A) Number and (B) length of neurites of PC12 cells cultured with BMDM supernatants in the five groups. BMDMs, bone marrow-derived macrophages. All the data were analyzed using one-way ANOVA followed by Tukey's post hoc test and are presented as the means ± SDs. $**p < 0.01$, $*p < 0.05$, and ** indicate statistical significance between the indicated groups; n = 6.

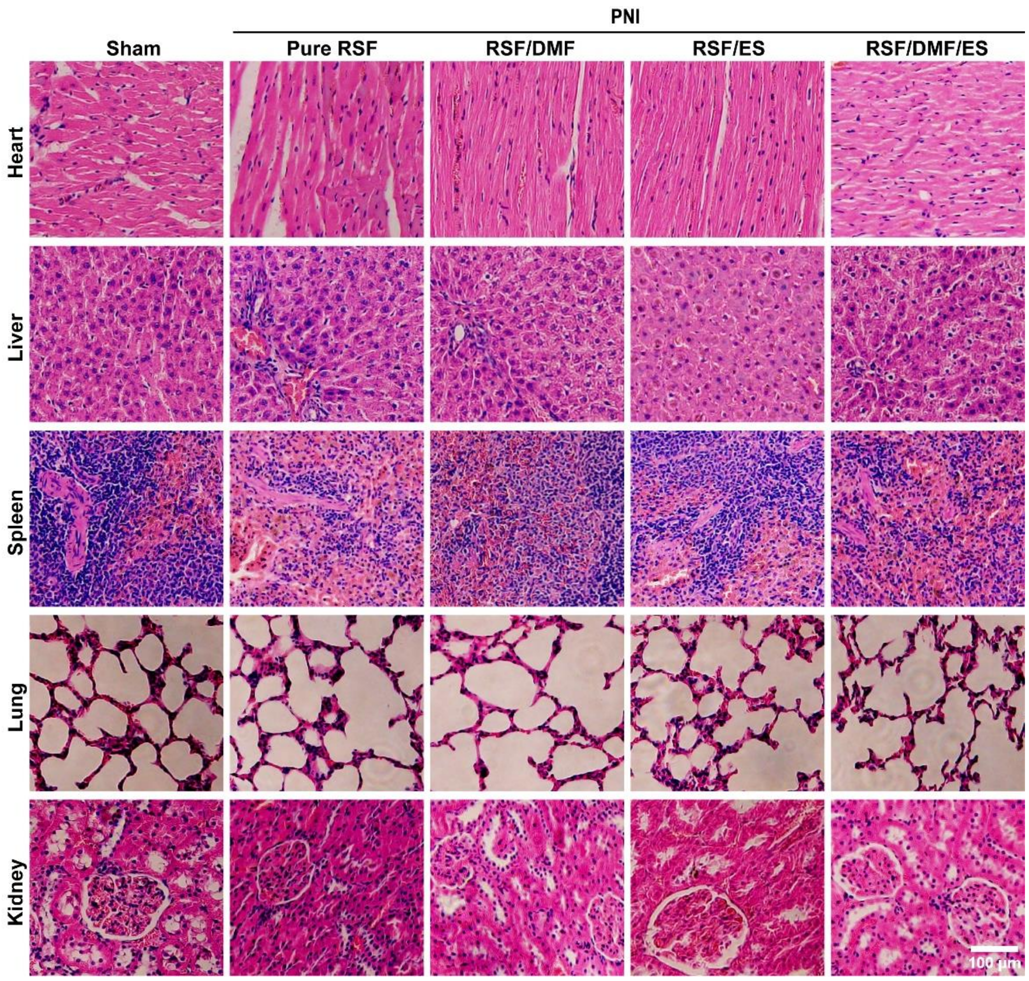

Figure S14. H&E staining of major organs (heart, liver, spleen, lungs, and kidneys) was performed to evaluate the biocompatibility of the DMF/RSF/P:P conduit. n = 6.

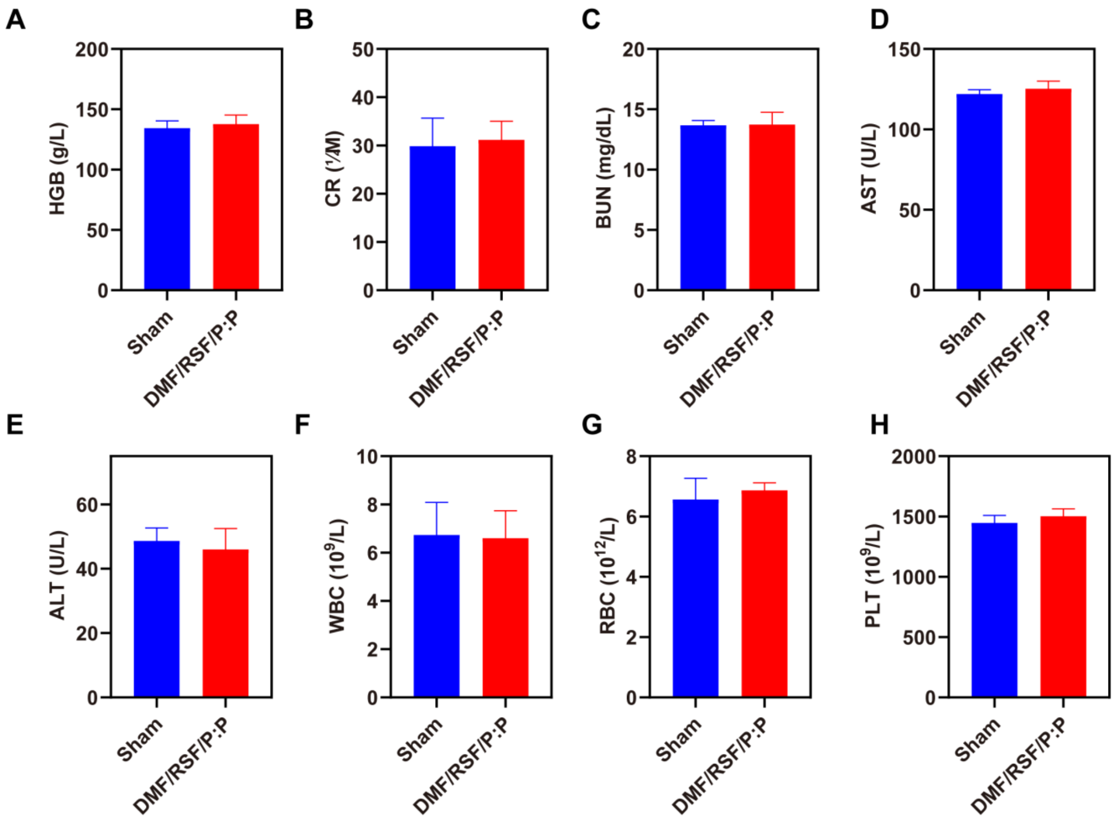

Figure S15. Biosafety evaluation of DMF/RSF/P:P conduit implantation 3 months postoperatively *in vivo*. (A–H) Blood routine and biochemical analysis results for evaluating the biosafety of the DMF/RSF/P:P conduit. The data are expressed as the mean ± standard deviation (S.D.). $^{**}p < 0.01$, $^{*}p < 0.05$, n = 6.

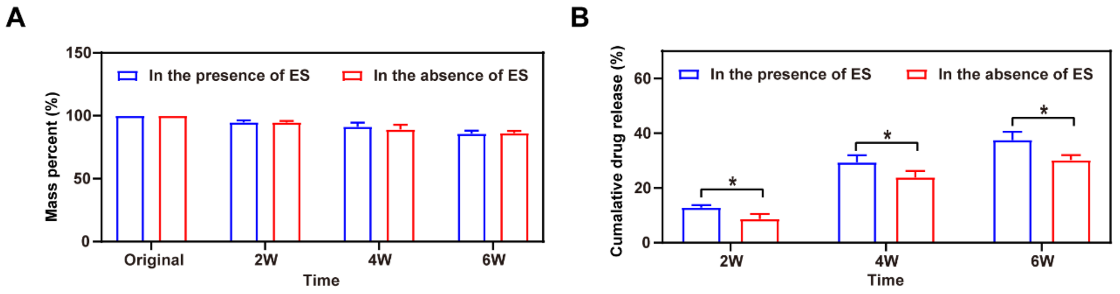

Figure S16. The effects of electrical stimulation (ES) on material performance. (A) Effect of electrical stimulation on the degradability of current NGCs. (B) Effect of electrical stimulation on the release of DMF. All the data were analyzed using one-way ANOVA followed by Tukey's post hoc test and are presented as the means ± SDs. **p < 0.01, *p < 0.05, and ** indicate statistical significance between the indicated groups; n = 3.

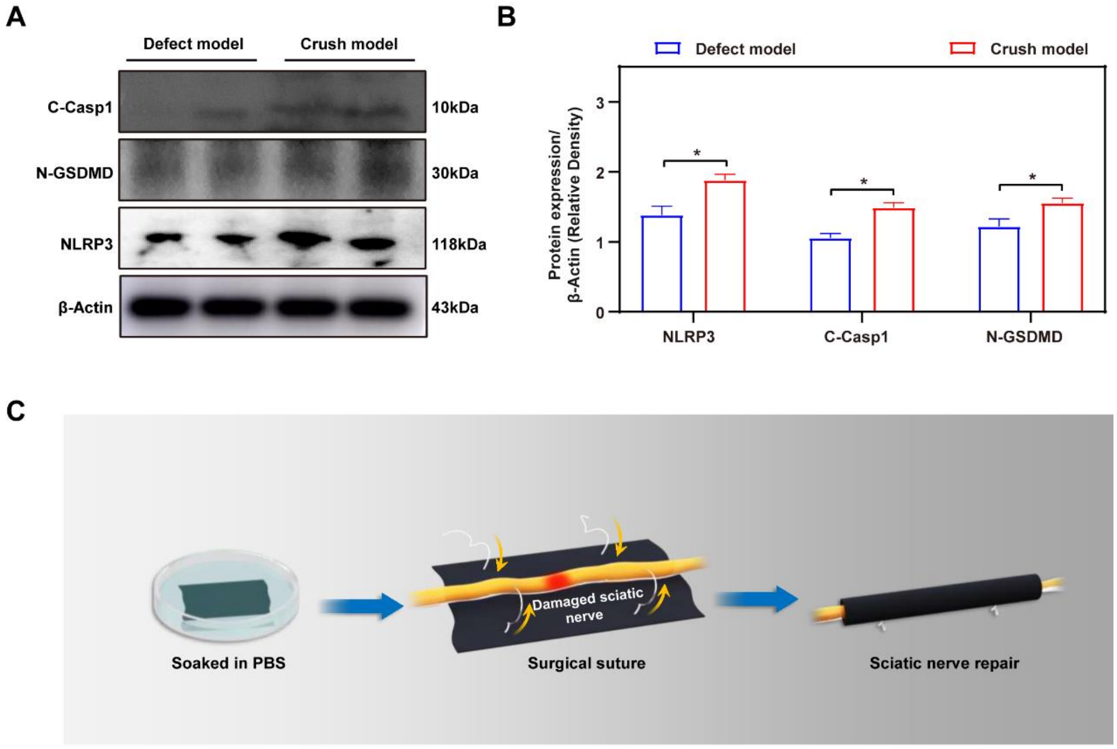

Figure S17. The establishment of the animal model in this study. (A-B) Protein levels and quantitative analysis of the pyroptosis-related proteins NLRP3, N-GSDMD, and C-Casp1 in nerve defect and crush model on the 3rd postoperative day. β-Actin was used as an internal control. The comparison of the pyroptosis phenomenon in the sciatic nerve crush model and defect model. (C) Scheme of the implantation of DMF/RSF/P:P conduits. All the data were analyzed using one-way ANOVA followed by Tukey's post hoc test and are presented as the means ± SDs. ***p* < 0.01 compared to the defect model. **p* < 0.05 compared to the defect model. n = 3.

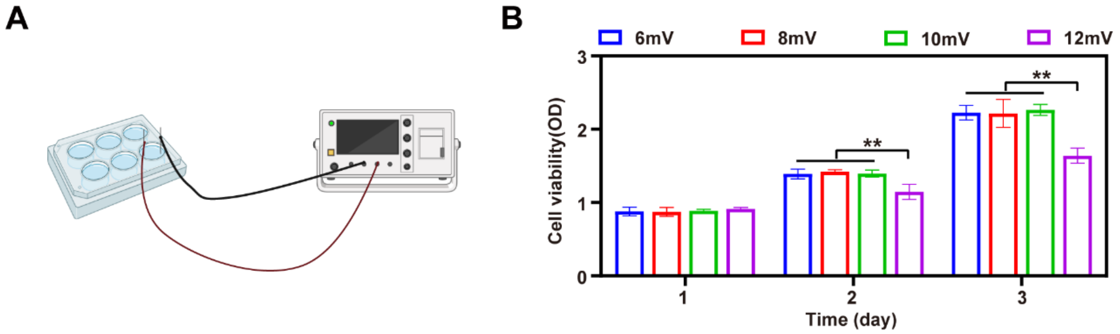

Figure S18. Electrical stimulation *in vitro*. (A) Scheme of electrical stimulation *in vitro*. (B) The determination of experimental parameters. All data were analyzed using one-way ANOVAs followed by Tukey's post-hoc test and are presented as mean ±SD. $^{**}p < 0.01$, $^{*}p < 0.05$, * and ** indicate statistical significance between indicated groups, n = 3.

Table S1 Primer sequences used for Real-time PCR.

Name	Sequence
CIL-6-F	5′- CTTCCCTACTTCACAAGTC - 3′
IL-6-R	5′- CTCCATTAGGAGAGCATTG - 3′
IL-8-F	5′- GCTGGAGCAAAAGGTATGGC - 3′
IL-8-R	5′- TGATCAGCTTCACCCAGGGA - 3′
NLRP3-F	5′- CTCTGCATGCCGTATCTGGT - 3′
NLRP3-R	5′- GACGTGCATGCATCATTCCA - 3′
iNos-F	5′- CTGCAGCCTCGCTACTACTC - 3′
iNos-R	5′- GGAGCTGAAAACCTCATCTGC - 3′
Arg1-F	5′- ATGTGCCCTCTGTCTTTTAGG - 3′
Arg1-R	5 - GTCCTGAAAGTAGCCCTGTCT - 3′
β-Actin-F	5′- CATGTACGTTGCTATCCAGGC - 3′
β-Actin-R	5′- CTCCTTAATGTCACGCACGAT - 3′
